# Trajectories of Adherence to Biologics in Patients With Inflammatory Bowel Diseases: A Large‐Scale Multi‐Regional Italian Study Through the VALORE Distributed Database

**DOI:** 10.1002/pds.70230

**Published:** 2025-10-09

**Authors:** Sabrina Giometto, Andrea Spini, Giorgia Pellegrini, Ylenia Ingrasciotta, Chiara Bellitto, Federica Soardo, Luca L'Abbate, Olivia Leoni, Arianna Mazzone, Domenica Ancona, Paolo Stella, Anna Cavazzana, Angela Scapin, Sara Lopes, Valeria Belleudi, Stefano Ledda, Paolo Carta, Paola Rossi, Lucian Ejlli, Ester Sapigni, Aurora Puccini, Alessandra Allotta, Sebastiano Addario Pollina, Roberto Da Cas, Giampaolo Bucaneve, Antea Maria Pia Mangano, Francesco Balducci, Carla Sorrentino, Ilenia Senesi, Marco Tuccori, Rosa Gini, Stefania Spila Alegiani, Marco Massari, Gianluca Trifirò, Ersilia Lucenteforte

**Affiliations:** ^1^ Department of Clinical and Experimental Medicine University of Pisa Pisa Italy; ^2^ Department of Diagnostics and Public Health University of Verona Verona Italy; ^3^ Lombardy Regional Epidemiologic Observatory Milan Italy; ^4^ Azienda Regionale per l'Innovazione e gli Acquisti, S.p.A Milan Italy; ^5^ Centro Regionale Farmacovigilanza Regione Puglia Bari Italy; ^6^ Azienda Zero, Regione Veneto Padova Italy; ^7^ Department of Epidemiology Lazio Regional Health Service Rome Italy; ^8^ Regione Autonoma Della Sardegna Cagliari Italy; ^9^ Friuli‐Venezia Giulia Regional Center of Pharmacovigilance Trieste Italy; ^10^ Emilia‐Romagna Regional Center of Pharmacovigilance Bologna Italy; ^11^ Epidemiologic Observatory of the Sicily Regional Health Service Palermo Italy; ^12^ Italian National Institute of Health Rome Italy; ^13^ Umbria Regional Centre of Pharmacovigilance Perugia Italy; ^14^ Agenzia Regionale Sanitaria Della Regione Marche Ancona Italy; ^15^ Regional Pharmaceutical Unit, Abruzzo Region Pescara Italy; ^16^ Abruzzo Regional Centre of Pharmacovigilance Teramo Italy; ^17^ Agenzia Regionale di Sanità Toscana Firenze Italy; ^18^ Department of Statistics, Computer Science and Applications “G. Parenti” University of Florence Florence Italy

**Keywords:** adherence, biological drugs, inflammatory bowel diseases, pharmacoepidemiology, trajectories

## Abstract

This study aimed to identify and describe trajectories of adherence to biologics in patients with IBDs and to identify adherence determinants in the Italian real‐world setting. We conducted a retrospective cohort study across 12 Italian regions, including new users of biologics with inflammatory bowel diseases (IBDs), between 2010 and 2019 and followed them for 3 years. We assessed adherence longitudinally, and we identified trajectories using nonparametric methods. To identify determinants of adherence, we used multinomial multivariate regression models. We included 20 150 subjects in the final cohort, mostly male (56%), < 65 years old (92%), and with Crohn's disease (58%). We identified three trajectories of adherence to biologics for IBDs: one group (19% of the cohort) maintained high adherence throughout the observation period, while the largest group (46%) initially reduced adherence, stabilizing around 70%. The remaining group (35%) gradually decreased adherence over the entire follow‐up, reaching 20%. Being female (odds ratio (OR) 1.52, 95% confidence interval (CI) 1.40–1.65), older (OR 1.44, 95% CI 1.21–1.70), and having adalimumab as index drug were each positively associated with low adherence compared to high adherence. In contrast, starting treatment with a biosimilar (OR 0.47, 95% CI 0.42–0.52) was negatively associated with low adherence. Our findings highlight that one in three patients with IBDs gradually reduced adherence to biologics within the first 3 years of treatment. Differences were observed according to the initial biologic dispensed and patient characteristics such as sex and age, with females and older patients positively associated with low adherence.

**Summary**

- New users of biologics for IBD show different long‐term adherence.
- Drug utilization differences depend on sex and age.
- Biosimilars have higher adherence compared to original biologics.
- Initial drug choice impacts adherence behavior.

## 1 Introduction

Inflammatory bowel diseases (IBDs) are a group of disorders characterized by chronic or relapsing inflammation of the gastrointestinal tract [1]. The two main pathologies are Crohn's disease (CD) and ulcerative colitis (UC). IBD conditions are lifelong and progressive, with higher mortality in CD patients and reduced quality of life [2]. Global prevalence increased from 79.5 per 100,000 in 1990 to 84.3 in 2017 [3]. A curative medical therapy is still not available for patients with these conditions. Therefore, pharmacological treatment aims to induce early remission and prevent disease relapse [4]. Alongside conventional drugs such as amino‐salicylates, corticosteroids, and DMARDs such as azathioprine, mercaptopurine, and methotrexate, biologic treatments including tumor necrosis factor (TNF)‐alpha inhibitors (adalimumab, infliximab, and golimumab), the anti‐interleukin (anti‐IL) ustekinumab, and the anti‐integrin vedolizumab have been available for over 20 years [5, 6]. Biologics have transformed the management of IBDs, offering effective options for patients with moderate to severe disease or those who fail traditional therapies. Their targeted action provides better disease control, improved quality of life, and a reduced need for surgery [7]. In chronic diseases, non‐adherence to drug therapy is a major obstacle to achieving patient health goals and improving clinical outcomes [8]. The proportion of days covered (PDC) and the medication possession ratio (MPR) are commonly used metrics to measure adherence. These metrics condense adherence into a single value, often dichotomized using predefined thresholds, and may not fully capture variation over time [9].

Previous studies assessing adherence to biological therapies in patients with IBD have used a variety of metrics. Adherence was often reported as the proportion of patients reaching a specific threshold of PDC, MPR, or mMPR [10, 11, 12, 13, 14, 15, 16], as average or median values of MPR or PDC [17, 18], or alternatively using metrics such as the continuous medication gap (CMG) [19], or the cumulative amount of time with a refill gap ≥ 20% (CG20) beyond the expected infusion interval [13].

Overall, these studies reported heterogeneous adherence estimates, with a proportion of adherent patients ranging from 41% to over 90%, depending on the adherence metric, threshold, and follow‐up. However, these studies did not account for behavioral changes in adherence over time.

To the best of our knowledge, only one study to date has evaluated adherence patterns longitudinally, reporting that more than one‐third of patients were classified within the consistent adherence group [20]. However, this study included a relatively small sample (approximately 300 individuals) and was limited to patients receiving hospital‐based intravenous therapies (infliximab and vedolizumab).

Adherence is a dynamic process characterized by alternating phases and degrees of adherence and non‐adherence during treatment initiation, maintenance, and discontinuation phases. As such, approaches based on a single summary measure may not fully reflect the complexity of adherence behavior.

To address these limitations, we built trajectories to describe longitudinal patterns of adherence over time [21].

Therefore, the aim of this study was to identify and describe trajectories of adherence to biologics in patients with IBDs in a large‐scale Italian real‐world setting. The secondary objective was to identify determinants of low adherence to biologics.

## 2 Materials and Methods

This study was a population‐based, multiregional, retrospective, descriptive cohort study. The study, which is part of the post‐marketing surveillance VALORE project [22], was approved by the Ethical Committee of the Academic Hospitals of Messina and Verona.

### 2.1 Data Sources

Data were locally extracted, converted into a common data model (CDM), and elaborated using a unique analytical script shared by the reference center to each participating Italian region: Abruzzo, Emilia‐Romagna, Friuli Venezia Giulia, Lazio, Lombardy, Marche, Puglia, Sardegna, Sicily, Tuscany, Umbria, and Veneto, which correspond to a population of almost 44 million, covering 74% of the Italian population from the North to the South and Islands [22, 23]. The data flow—from raw regional data to the analytical dataset—is depicted in the first figure of the original Valore project paper [22]. The process of data elaboration, quality check, matching, and record linkage, as well as the creation of variables, was conducted using the open‐source tool ‘TheShinISS’ for the conduction of distributed analyses in pharmacoepidemiologic multi‐database studies, developed using R software by researchers of the Italian National Institute of Health [24]. The analysis of this study was programmed in R as a module for TheShinISS and distributed to the regional partners to be run locally. The output was an analytical dataset to be shared with the University of Verona [25]. Collected data included: demographic information (date of birth, sex, and date of registration in the regional healthcare system) from the inhabitant registry; drug dispensing, coded using the anatomical therapeutic chemical (ATC) classification system and national drug code, from pharmacy claims databases; hospital information, including date of admission and discharge, main diagnosis, and up to five secondary diagnoses coded using the International Classification of Diseases, Ninth Revision, Clinical Modification (ICD‐9‐CM), and principal procedure and up to five secondary procedures, from hospital discharge records; exemptions from co‐payments, including coded information about chronic diseases; outpatient diagnostic tests and specialist visits database; drug prescription, including the exact drug name, number of dispensed packages, and dosing regimen.

A graphical representation of the study design is shown in Figure S1.

### 2.2 Study Population and Cohort Selection

All subjects residing in the catchment area of the included regions during the years 2010–2022 (Sardegna from 2012, Puglia from 2014, Lazio up to 2020) were identified. All subjects with at least one biologic dispensing approved for the treatment of IBDs between January 1st, 2010 and December 31st, 2022 (or the last available date) were selected. The following biologics (both originators and biosimilars, if available on the market) were considered: adalimumab (ATC code L04AB04), infliximab (L04AB02), golimumab (L04AB06), vedolizumab (L04AA33), and ustekinumab (L04AC05). Of note, ustekinumab users were considered only if the dispensation date was after September 1st, 2018, as this biologic received an IBD indication on that date. The first date of the biologic dispensing was defined as the index date, and the corresponding drug was defined as the index drug. Among biologic users, those with at least 1 year of observation before the index date were included, and only subjects with no dispensing of biologics in the year before the index date were considered (i.e., new‐user). Moreover, subjects with less than 3 years of observation after the index date (follow‐up) were excluded to allow a long‐term treatment adherence evaluation for all the subjects included in the cohort: thus, subjects included in 2020, 2021, and 2022 were excluded by definition. Of these, all patients with IBDs (i.e., CD and UC) indication were eligible for the study cohort. Indication of use was retrieved using a published validated algorithm with high diagnostic accuracy (sensitivity, specificity, positive, and negative predictive value above 80%, except for PPV in UC) [26].

### 2.3 Variables

We calculated monthly adherence to biologics for each subject. Since we observed subjects for 3 years after the index date, we had 36 adherence measures for each patient included in the study cohort. To estimate monthly biologics adherence, we calculated the PDC, i.e., we divided the number of days covered by at least one biologic drug by the number of days of the month (28–31 days, as appropriate). In the PDC measurement, days covered by more than one dispensing were counted only once to avoid exposure overestimation (e.g., loading doses of biologics). The duration of a single dispensation—that is, the period during which an individual was considered to be on treatment based on that dispensation—was estimated by assuming a daily intake of one defined daily dose (DDD) (Table S1).

We considered the following covariates: (1) sex and age at the index date (ID); (2) comorbidities in the whole period available in the databases prior to ID: hypertension, atrial fibrillation, ischemic heart disease, cerebrovascular disease, diabetes mellitus, chronic kidney disease, chronic pulmonary disease, liver disease, neoplasms, and other immune‐mediated inflammatory diseases (IMIDs) (see Table S2); and (3) drug therapies dispensed within the year before the index date: antiplatelet drugs, anti‐arrhythmic drugs, anticoagulants, antibacterials for systemic use, antivirals for systemic use, antidepressants, antipsychotics, conventional synthetic DMARDs, glucocorticoids for systemic use, non‐steroidal anti‐inflammatory drugs (NSAIDs), and drugs for peptic ulcer and gastro‐esophageal reflux disease (Table S3). We considered those covariates because we believe they may be associated with treatment adherence, as patients with comorbidities are expected to be less adherent. Among the comorbidities, we selected chronic and serious conditions that may affect the patient's overall health status. In addition, we considered several treatments received prior to the biologic, as polytherapy is known to influence treatment discontinuation [27].

In the follow‐up period, the following variables were also defined: (1) non‐biological drug therapies, with and without indication for IBDs (same drugs listed in Table S3) and (2) any dispensation of biologic different from the index drug, with a different active substance or a different type (e.g., biosimilar, originator) (switch) [28].

### 2.4 Statistical Analysis

To define trajectories of adherence, we used the method elaborated by Leffondrè et al. [21], which is a three‐step procedure:

1. The computation of 24 statistical measures, which summarize the variability of adherence for each subject. These measures could be computed only for subjects with at least three consecutive adherence values. Each of these measures highlights a specific aspect of variability, and they can be grouped into four classes: (1) basic descriptive statistics, such as range or standard deviation, that do not take into account the temporal sequence of the scores; (2) elementary measures of change, such as the difference between the last and the first score, that gives information on the progression between the first and the last score, but do not discriminate between subjects with different patterns; (3) measures of nonlinearity and of the inconsistency of changes, based on the first differences, that are the changes between two consecutive scores of the same subject; these measures can identify increases/decreases between two scores, for a subject; and (4) measures of nonmonotonicity, based on the second differences, that are the differences between two consecutive first differences; they identify also abrupt fluctuations that are large increase/decrease followed by large decrease/increase, respectively.
2. Principal component analysis, which selects the subset of measures that explain the largest proportion of variance in data. A combination of all measures would completely describe the patterns of change, but it would give problems of redundancy and therefore of interpretability. Thus, a selection of measures was necessary;
3. Cluster analysis, based on the selected previously standardized measures, which groups patients with similar longitudinal trajectories. The method adopted to perform the cluster analysis was the *k*‐means algorithm, which consists of an iterative system for assigning the observations to the nearest cluster by choosing as initial centroid of the *k* clusters, *k* random points, and modifying these centroids at each iteration, recalculating the average value for each cluster. The number of clusters *k* is needed a priori, to perform the *k*‐means method. There is no consensus regarding the best and the optimal method for choosing the number of clusters. We considered a combination of a mathematical criterion, consisting of the calculations of 30 indexes based on measures of distance and considering the majority rule, and a clinical plausibility criterion.

We graphically represented the adherence trajectories as monthly average PDC values over the 3‐year follow‐up period. We then stratified the cohort by condition and conducted the analysis within the two groups.

To identify the determinants of low adherence, we fitted a multinomial regression model, selecting variables from all those available through a stepwise algorithm based on the Akaike information criterion (AIC). We reported the model results in terms of OR and their 95% confidence interval (CI) graphically in a forest plot.

To better describe adherence behavior, we compared the use of biological (switch) and non‐biological drugs during the 3 years of observation after ID across clusters.

Finally, we conducted a secondary analysis identifying the adherence trajectories and their determinants over a period of 5 years of observation after the index date.

The analyses were conducted using R software, version 4.3.2, and trajectory analysis using the traj package, version 2.0.1 (code freely available on GitHub [29]).

## 3 Results

**FIGURE 1:**
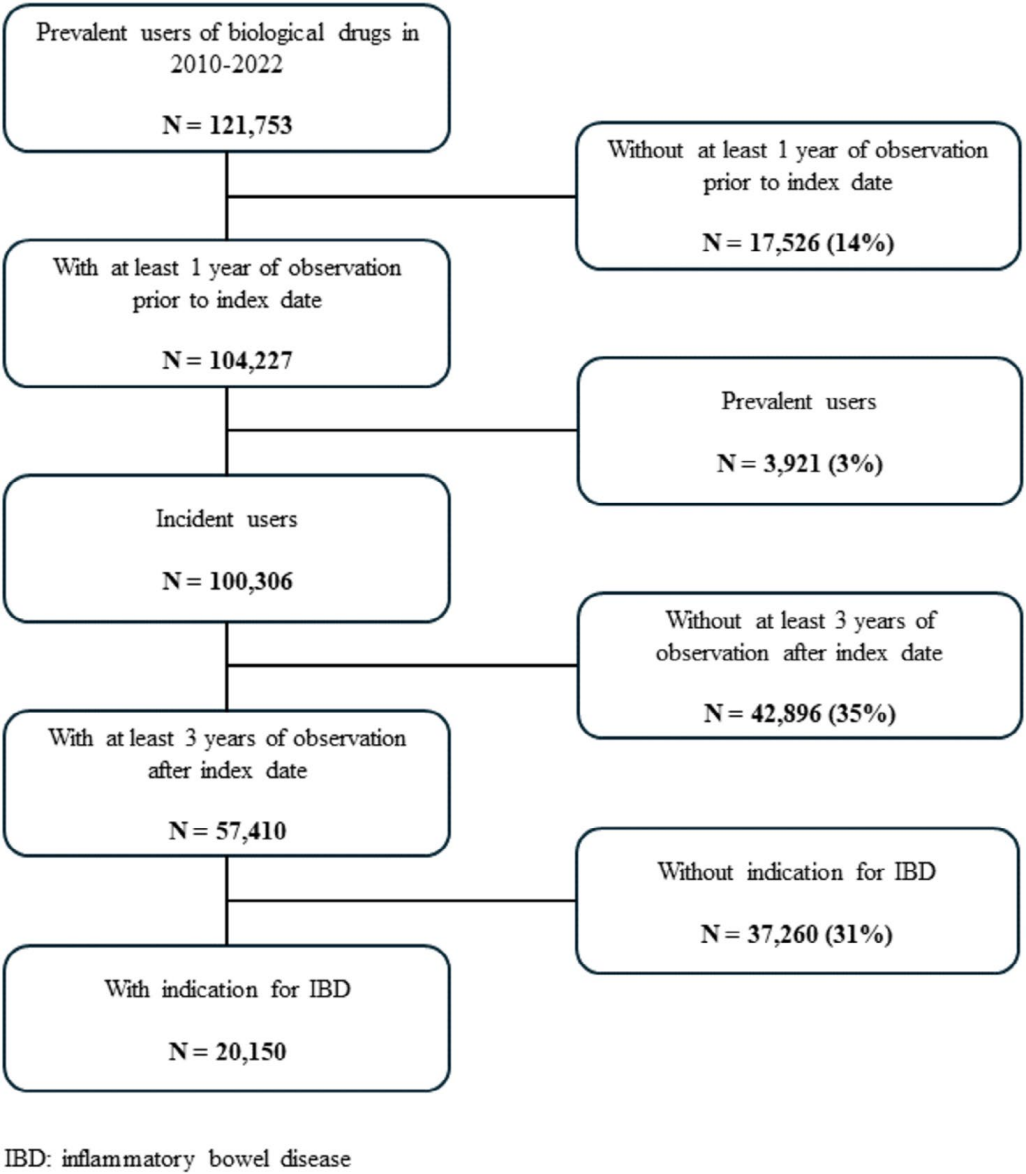
Flowchart of patient selection and cohort identification for the study analysis, showing inclusion and exclusion criteria with the corresponding numbers and percentages at each step.

We included in the analysis 20 150 new users of biologics who were treated because of IBDs from 12 Italian regions (Abruzzo: 415 (2.1%), Emilia‐Romagna: 2068 (10.3%), Friuli‐Venezia Giulia: 443 (2.2%), Latium: 1841 (9.1%), Lombardy: 3463 (17.2%), Marche: 1038 (5.2%), Apulia: 2773 (13.8%), Sardinia: 542 (2.7%), Sicily: 2992 (14.8%), Tuscany: 1801 (8.9%), Umbria: 493 (2.4%), and Veneto: 2278 (11.3%)) (Figure 1).

We observed a higher frequency of males (55.5%), patients younger than 65 years (92.0%), with biologics mostly administered for CD (58.2%) (Table 1). Biologics more frequently dispensed as index drugs were adalimumab and infliximab (47.3% and 41.2%, respectively). We observed intestinal infections and hypertension as the most frequent comorbidities during the look‐back period (11.5% and 8.9%, respectively).

**TABLE 1:** Distribution of cohort characteristics at and before index date.

	Overall (*N* = 20 150)	Crohn's disease (*N* = 11 720)	Ulcerative colitis (*N* = 8430)
*At index date*			
Sex, males, *N* (%)	11 184 (55.5)	6422 (54.8)	4762 (56.4)
Age, *N* (%)			
< 18	1234 (6.1)	792 (6.8)	442 (5.2)
18–44	10 025 (49.8)	6049 (51.6)	3976 (47.2)
45–64	7258 (36.0)	4050 (34.6)	3208 (38.1)
65–79	1568 (7.8)	803 (6.9)	765 (9.1)
≥ 80	65 (0.3)	26 (0.2)	39 (0.5)
Index drug, *N* (%)			
Adalimumab	9540 (47.3)	7013 (59.8)	2527 (30.0)
Golimumab	991 (4.9)	154 (1.3)	837 (9.9)
Infliximab	8307 (41.2)	3981 (34.0)	4326 (51.3)
Ustekinumab	83 (0.4)	81 (0.7)	2 (0.0)
Vedolizumab	1229 (6.1)	491 (4.2)	738 (8.8)
Index drug route of administration, *N* (%)			
Intravenous	9575 (47.5)	4510 (38.5)	5065 (60.1)
Subcutaneous	10 575 (52.5)	7210 (61.5)	3365 (39.9)
Index class, *N* (%)			
Anti‐interleukins	83 (0.4)	81 (0.7)	2 (0.0)
Selective immunosuppressant	1229 (6.1)	491 (4.2)	738 (8.8)
Tumor necrosis factor‐alpha inhibitors	18 838 (93.5)	11 148 (95.1)	7690 (91.2)
Type of index drug, *N* (%)			
Biosimilar	4507 (22.4)	2286 (19.5)	2221 (26.3)
Originator	15 643 (77.6)	9434 (80.5)	6209 (73.7)
*Before index date (ever)*			
History of comorbidity, *N* (%)			
Hypertension	1803 (8.9)	898 (7.7)	905 (10.7)
Atrial fibrillation	161 (0.8)	77 (0.7)	84 (1.0)
Ischemic heart disease	340 (1.7)	150 (1.3)	190 (2.3)
Cerebrovascular disease	230 (1.1)	121 (1.0)	109 (1.3)
Diabetes mellitus	830 (4.1)	356 (3.0)	474 (5.6)
Chronic renal disease	195 (1.0)	110 (0.9)	85 (1.0)
Chronic liver disease	204 (1.0)	104 (0.9)	100 (1.2)
Chronic pulmonary disease	192 (1.0)	115 (1.0)	77 (0.9)
History of tumors	937 (4.7)	520 (4.4)	417 (4.9)
Intestinal infections	2313 (11.5)	1690 (14.4)	623 (7.4)
Other IMIDs			
Psoriasis	934 (4.6)	540 (4.6)	394 (4.7)
Psoriatic arthritis	621 (3.1)	354 (3.0)	267 (3.2)
Rheumatoid arthritis	515 (2.6)	281 (2.4)	234 (2.8)
Ankylosing spondylitis	896 (4.4)	546 (4.7)	350 (4.2)
*Before index date (1 year)*			
Previous use of drugs, *N* (%)			
Antiplatelet agents	67 (0.3)	40 (0.3)	27 (0.3)
Antiarrhythmics	15 (0.1)	10 (0.1)	5 (0.1)
Anticoagulants	692 (3.4)	522 (4.5)	170 (2.0)
Antidepressants	188 (0.9)	89 (0.8)	99 (1.2)
Antipsychotics	45 (0.2)	25 (0.2)	20 (0.2)
Antibacterial for systemic use	111 (0.6)	68 (0.6)	43 (0.5)
Antivirals for systemic use	241 (1.2)	123 (1.0)	118 (1.4)
Drugs for peptic ulcer and gastro‐esophageal reflux disease	636 (3.2)	353 (3.0)	283 (3.4)
csDMARDs	1396 (6.9)	896 (7.6)	500 (5.9)
Methotrexate	190 (0.9)	139 (1.2)	51 (0.6)
Sulfasalazine	256 (1.3)	146 (1.2)	110 (1.3)
Mesalazine	975 (4.8)	633 (5.4)	342 (4.1)
Balsalazide	17 (0.1)	5 (0.0)	12 (0.1)
Glucocorticoids	389 (1.9)	255 (2.2)	134 (1.6)
NSAIDs	326 (1.6)	177 (1.5)	149 (1.8)

*Note:* Distribution of cohort characteristics at index date (demographics, index drug, and type of index drug), before the index date (history of comorbidities and previous drug use), overall and by indication for use.

Abbreviations: csDMARDs, conventional synthetic disease‐modifying antirheumatic drugs; NSAIDs, nonsteroidal anti‐inflammatory drugs.

The distribution of cohort characteristics by index drug is reported in Table S4.

**FIGURE 2:**
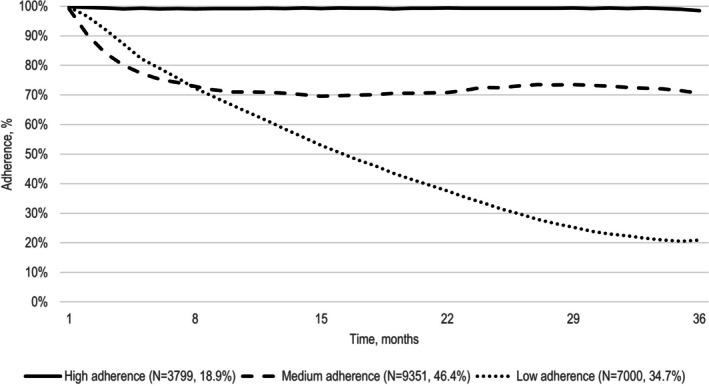
Trajectories of adherence to biologics in IBD patients new users of the drug in the VALORE network, which includes a population of almost 44 million, covering 74% of the Italian population from the North to the South and Islands. The figure shows treatment adherence trajectories over time (months on the *x*‐axis, adherence percentage on the *y*‐axis). Three distinct patterns of adherence were identified, with the corresponding numbers and percentages of patients reported for each.

**FIGURE 3:**
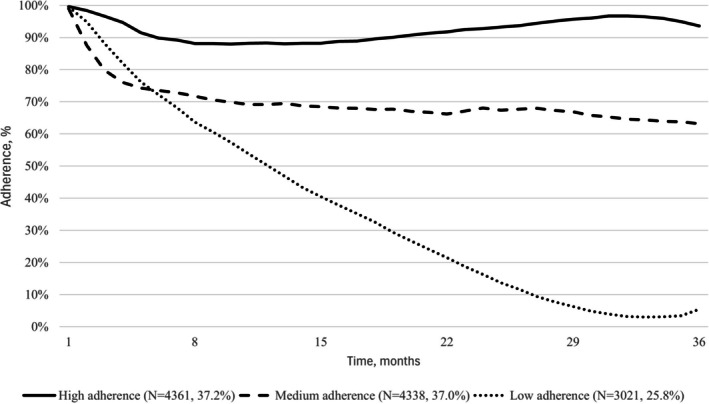
Trajectories of adherence to biological drugs in patients with Crohn's disease new users of the drug in the VALORE network, which includes a population of almost 44 million, covering 74% of the Italian population from the North to the South and Islands. The figure shows treatment adherence trajectories over time (months on the *x*‐axis, adherence percentage on the *y*‐axis). Three distinct adherence patterns were identified, with the corresponding numbers and percentages of patients reported for each.

**FIGURE 4:**
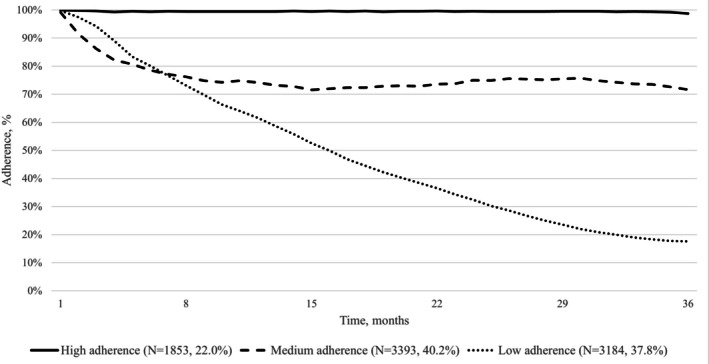
Trajectories of adherence to biological drugs in patients with ulcerative colitis new‐users of the drug in the VALORE network, which includes a population of almost 44 million, covering 74% of the Italian population from the North to the South and Islands. The figure shows treatment adherence trajectories over time (months on the *x*‐axis, adherence percentage on the *y*‐axis). Three distinct adherence patterns were identified, with the corresponding numbers and percentages of patients reported for each.

We observed three trajectories of adherence to biologics for IBDs over a 3‐year follow‐up period (Figure 2). A first smaller group, which includes 19% of the subjects in the cohort, shows high adherence throughout the entire observation period; the largest group (46%) shows a reduction in the first months of use, followed by a stable trend with around 70% treatment adherence level; the last one includes 35% of subjects and shows a gradual reduction over the entire follow‐up period, up to values of 20% treatment adherence. Stratifying by disease, we observed for CD a smaller group of low‐adherents (26%), who showed a gradual reduction up to values close to zero (Figure 3). Instead, we observed for UC a larger group of low‐adherents (38%) who, as in the overall analysis, showed adherence values of 20% at the end of the 3 years of follow‐up (Figure 4).

We observed a higher frequency of males, infliximab as the index drug, and biosimilar as the index drug type among highly adherent users (Table S5). Instead, among low adherents, we observed a higher frequency of females and older individuals. Adalimumab as an index drug and CD as an indication of use were more frequent among medium adherents. For the Crohn's group, we observed the same distribution as in the overall analysis (Table S6). Instead, for the UC group, we observed a higher frequency of infliximab as the index drug among low adherents (Table S7). However, it should be noted that the index drug mostly used among all individuals with CD was adalimumab (60%), followed by infliximab (34%). Conversely, in the UC group, infliximab was the most dispensed (51%), followed by adalimumab (30%).

**FIGURE 5:**
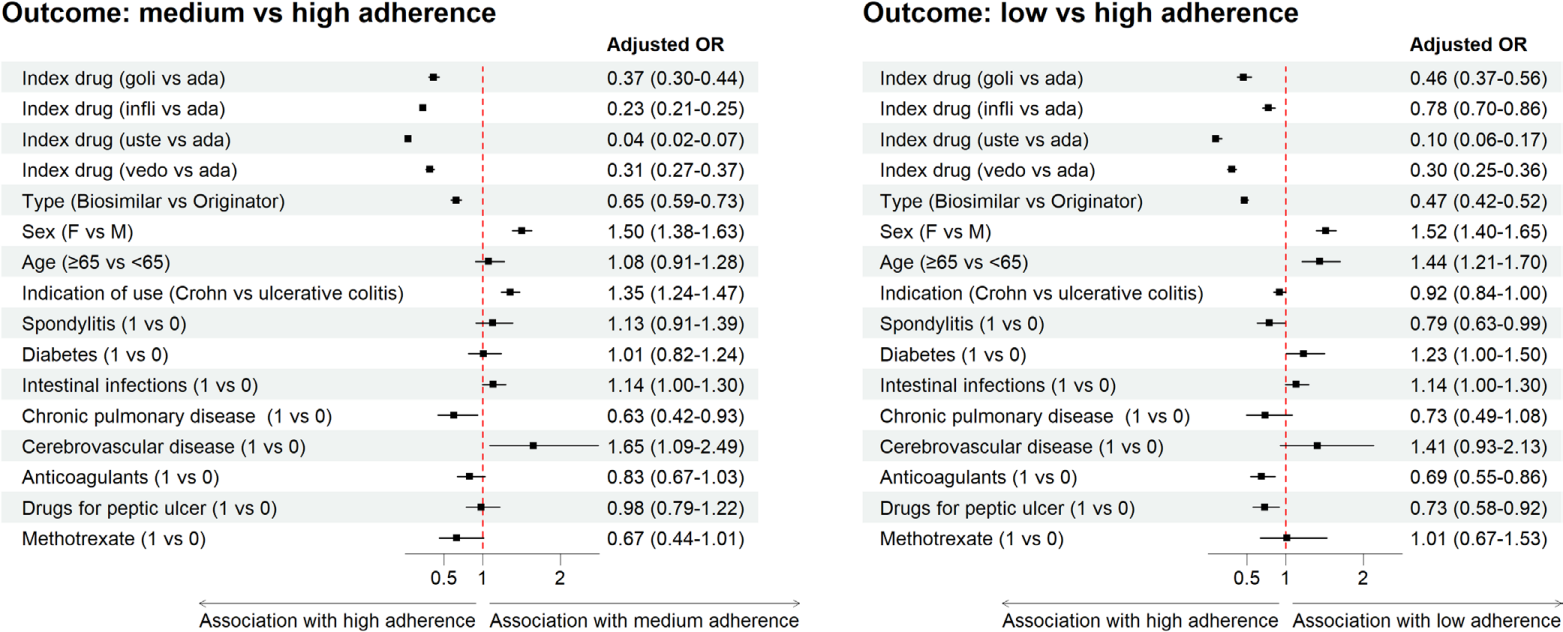
Determinants of treatment adherence to biologics at 3 years of follow‐up. Forest plots reporting adjusted odds ratios (OR) and 95% confidence intervals (CI) from the multinomial regression model. The black squares represent the point estimates of the odds ratio, while the horizontal bars indicate the width of the confidence intervals. The dashed red line corresponds to the line of null effect and is centered on the value of 1 (no effect: The odds of medium or low adherence are equal in group A (e.g., Golimumab) and group B (e.g., Adalimumab)). Squares to the right of the line of indifference indicate a positive association of the corresponding variable (or level of it) with medium/low adherence (medium in the left graph, low in the right). Squares to the left of the line of indifference indicate a positive association of the corresponding variable (or level of it) with high adherence.

In the multivariate analysis, the variables included in the model according to the AIC were: sex, age, index drug, index type, indication of use, spondylitis, diabetes mellitus, intestinal infections, chronic pulmonary disease, cerebrovascular disease, as well as use of anticoagulant, drugs for peptic ulcer 1 year prior ID, and methotrexate. All except for diabetes mellitus, intestinal infections, and use of methotrexate were identified as predictors of adherence to biological therapies in IBD patients (Figure 5). We also accounted for regional variability by adjusting the multinomial models for the region. However, for simplicity of reading, we did not report results on regional variability, even though we observed that this variable contributed to explaining the variability in adherence. Specifically, being female (adj. OR 1.52, 95% CI 1.40–1.65) and older (1.44, 95% CI 1.21–1.70) were positively associated with low adherence as compared to high adherence. Looking at index drugs, as compared to adalimumab, all other biologics approved for IBD (golimumab: 0.46, 95% CI 0.37–0.56; infliximab: 0.78, 95% CI 0.70–0.86; ustekinumab: 0.10, 95% CI 0.06–0.17; vedolizumab: 0.30, 95% CI 0.25–0.36) were negatively associated with low adherence. The same held true for the use of biosimilars as index drugs (0.47, 95% CI 0.42–0.52), having ankylosing spondylitis as comorbidity (0.79, 95% CI 0.63–0.99), and using anticoagulants (0.69, 95% CI 0.55–0.86) or drugs for peptic ulcer (0.73, 95% CI 0.58–0.92). Instead, CD as an indication for biologic use (1.35, 95% CI 1.24–1.47) and the presence of cerebrovascular disease (1.65, 95% CI 1.09–2.49) were positively associated with medium adherence as compared to high adherence, whereas age and the use of anticoagulants or drugs for peptic ulcer were not associated anymore. Instead, chronic pulmonary disease (0.63, 95% CI 0.42–0.93) was negatively associated with low adherence compared to high adherence.

Considering the follow‐up period (at 3 years after index date), low‐adherents used more drugs, likely due to the presence of multiple comorbidities. This higher drug use may, in turn, lead to potential complications—not necessarily specific to IBD, but in general, compared to medium and, especially, high adherents (anticoagulants: 12.0% vs. 7.7% and 4.0%, respectively; drugs for peptic ulcer and gastro‐esophageal reflux disease: 4.5% vs. 3.2% and 2.7%, respectively; antidepressants: 3.9% vs. 3.0% and 2.3%, respectively; antiplatelets: 1.5% vs. 0.8% and 0.9%, respectively). Occurrence of a switch at 3 years of follow‐up ranged from 45.5% (medium adherent) to 25.8% (low adherent subjects). Median time to first switch was 485 days in the overall cohort, with the lowest value among low adherents (346 days) and the highest among medium adherents (530 days) (Table 2). Few patients used a JAK‐inhibitor during the follow‐up period (18, 0.1%, data not shown). We observed in both Crohn's and UC groups the same distribution of drug use, with low‐adherents who used more drugs for possible complications compared to medium and, especially, high adherents (Tables 3 and 4).

**TABLE 2:** Distribution of non‐biological drugs and biological drugs (switch) users during follow‐up, overall and by cluster.

	Overall (*N* = 20 150)	High adherence (*N* = 3799)	Medium adherence (*N* = 9351)	Low adherence (*N* = 7000)
After index date (3 years)				
Antiplatelet agents (%)	216 (1.1)	33 (0.9)	76 (0.8)	107 (1.5)
Anticoagulant agents (%)	1712 (8.5)	151 (4.0)	718 (7.7)	843 (12.0)
Antiarrhythmics (%)	46 (0.2)	6 (0.2)	19 (0.2)	21 (0.3)
Antibacterial for systemic use (%)	278 (1.4)	63 (1.7)	121 (1.3)	94 (1.3)
Antivirals for systemic use (%)	862 (4.3)	163 (4.3)	392 (4.2)	307 (4.4)
Antidepressant drugs (%)	643 (3.2)	87 (2.3)	284 (3.0)	272 (3.9)
Antipsychotic drugs (%)	187 (0.9)	34 (0.9)	85 (0.9)	68 (1.0)
Drugs for peptic ulcer and gastro‐esophageal reflux disease (%)	712 (3.5)	101 (2.7)	297 (3.2)	314 (4.5)
csDMARDS (%)	1963 (9.7)	274 (7.2)	931 (10.0)	758 (10.8)
Balsalazide (%)	21 (0.1)	1 (0.0)	9 (0.1)	11 (0.2)
Mesalazina (%)	957 (4.7)	142 (3.7)	452 (4.8)	363 (5.2)
Methotrexate (%)	606 (3.0)	66 (1.7)	315 (3.4)	225 (3.2)
Sulfasalazine (%)	523 (2.6)	80 (2.1)	224 (2.4)	219 (3.1)
Glucocorticoids (%)	604 (3.0)	104 (2.7)	293 (3.1)	207 (3.0)
NSAIDs (%)	815 (4.0)	132 (3.5)	381 (4.1)	302 (4.3)
Switch	7301 (36.2)	1238 (32.6)	4255 (45.5)	1808 (25.8)
Time to first switch, median (IQR), days	485 (240, 759)	461 (216, 762)	530 (299, 768)	346 (166, 714)

*Note:* Distribution of non‐biological drugs and biological drugs (switch) users during follow‐up, overall and by cluster of treatment adherence.

Abbreviations: csDMARDs, conventional synthetic disease‐modifying antirheumatic drugs; NSAIDs, nonsteroidal anti‐inflammatory drugs; TNF, tumor necrosis factor.

**TABLE 3:** Distribution of non‐biological drugs and biological drugs (switch) users during follow‐up, overall and by cluster for Crohn's disease only.

	Overall (*N* = 11 720)	High adherence (*N* = 4361)	Medium adherence (*N* = 4338)	Low adherence (*N* = 3021)
After index date (3 years)				
Antiplatelet agents	110 (0.9)	35 (0.8)	31 (0.7)	44 (1.5)
Anticoagulants	1000 (8.5)	268 (6.1)	338 (7.8)	394 (13.0)
Antiarrhythmics	26 (0.2)	2 (0.0)	9 (0.2)	15 (0.5)
Antibacterial for systemic use	152 (1.3)	64 (1.5)	49 (1.1)	39 (1.3)
Antivirals for systemic use	475 (4.1)	175 (4.0)	177 (4.1)	123 (4.1)
Antidepressants	363 (3.1)	103 (2.4)	141 (3.3)	119 (3.9)
Antipsychotics	102 (0.9)	33 (0.8)	38 (0.9)	31 (1.0)
Drugs for peptic ulcer and gastro‐esophageal reflux disease	396 (3.4)	141 (3.2)	129 (3.0)	126 (4.2)
csDMARDs	1256 (10.7)	432 (9.9)	462 (10.7)	362 (12.0)
Balsalazide	8 (0.1)	2 (0.0)	5 (0.1)	1 (0.0)
Mesalazine	655 (5.6)	223 (5.1)	245 (5.6)	187 (6.2)
Methotrexate	398 (3.4)	141 (3.2)	143 (3.3)	114 (3.8)
Sulfasalazine	300 (2.6)	99 (2.3)	104 (2.4)	97 (3.2)
Glucocorticoids	417 (3.6)	152 (3.5)	140 (3.2)	125 (4.1)
NSAIDs	468 (4.0)	152 (3.5)	169 (3.9)	147 (4.9)
Switch	3975 (33.9)	1822 (41.8)	1619 (37.3)	534 (17.7)
Time to first switch, median (IQR), days	545 (294, 805)	559 (309, 795)	588 (336, 814)	360 (159, 714)

*Note:* Distribution of non‐biological drugs and biological drugs (switch) users during follow‐up among patients with Crohn's disease, overall and by cluster of treatment adherence.

Abbreviations: csDMARDs, conventional synthetic disease‐modifying antirheumatic drugs; NSAIDs, nonsteroidal anti‐inflammatory drugs; TNF, tumor necrosis factor.

**TABLE 4:** Distribution of non‐biological drugs and biological drugs (switch) users during follow‐up, overall and by cluster for ulcerative colitis only.

	Overall (*N* = 8430)	High adherence (*N* = 1853)	Medium adherence (*N* = 3393)	Low adherence (*N* = 3184)
After index date (3 years)				
Antiplatelet agents	106 (1.3)	19 (1.0)	35 (1.0)	52 (1.6)
Anticoagulants	712 (8.4)	78 (4.2)	222 (6.5)	412 (12.9)
Antiarrhythmics	20 (0.2)	4 (0.2)	9 (0.3)	7 (0.2)
Antibacterial for systemic use	126 (1.5)	30 (1.6)	54 (1.6)	42 (1.3)
Antivirals for systemic use	387 (4.6)	88 (4.7)	159 (4.7)	140 (4.4)
Antidepressants	280 (3.3)	47 (2.5)	105 (3.1)	128 (4.0)
Antipsychotics	85 (1.0)	21 (1.1)	36 (1.1)	28 (0.9)
Drugs for peptic ulcer and gastro‐esophageal reflux disease	316 (3.7)	55 (3.0)	107 (3.2)	154 (4.8)
csDMARDs	707 (8.4)	108 (5.8)	282 (8.3)	317 (10.0)
Balsalazide	13 (0.2)	0 (0.0)	3 (0.1)	10 (0.3)
Mesalazine	302 (3.6)	56 (3.0)	106 (3.1)	140 (4.4)
Methotrexate	208 (2.5)	23 (1.2)	106 (3.1)	79 (2.5)
Sulfasalazine	223 (2.6)	35 (1.9)	83 (2.4)	105 (3.3)
Glucocorticoids	187 (2.2)	47 (2.5)	85 (2.5)	55 (1.7)
NSAIDs	347 (4.1)	69 (3.7)	145 (4.3)	133 (4.2)
Switch	3326 (39.5)	651 (35.1)	1783 (52.5)	892 (28.0)
Time to first switch, median (IQR), days	399 (203, 700)	394 (195, 713)	468 (240, 715)	289 (151, 597)

*Note:* Distribution of non‐biological drugs and biological drugs (switch) users during follow‐up among patients with ulcerative colitis, overall and by cluster of treatment adherence.

Abbreviations: csDMARDs, conventional synthetic disease‐modifying antirheumatic drugs; NSAIDs, nonsteroidal anti‐inflammatory drugs; TNF, tumor necrosis factor.

We observed a lower frequency of users who switched in the Crohn's group (33.9%) compared to the UC group (39.5%), with a lower median time to first switch for the latter (399 days) compared to the Crohn's group (545 days).

The secondary analysis over a longer follow‐up period (5 years) revealed similar adherence trends, with the completely adherent group being smaller and the group gradually reducing treatment adherence over time, reaching values close to zero at the end of the 5 years (Figure S2). Additionally, it was observed that the use of antidepressants in the look‐back period was positively associated with low adherence (OR 2.67, 95% CI 1.12–6.37, Figure S3).

## 4 Discussion

To the best of our knowledge, this is the first population‐based study where adherence to biologics in patients with IBDs has been assessed longitudinally in the Italian real‐world setting.

We observed three distinct patterns of adherence to biologics in IBD patients over the initial 3 years of use. The largest group (46%) showed a decline in adherence within the first 9 months of treatment, reaching approximately 70% adherence after a 3‐year period. According to a widely used threshold of around 80% [30], this level of adherence would be considered slightly suboptimal. Approximately one‐third of the cohort gradually reduced treatment adherence over the first 3 years, reaching adherence levels around 20%, which, upon longer follow‐up, decreased to nearly zero after 5 years of use. Lastly, around 20% of the cohort showed a high adherence pattern over the initial 3 years of use, which persisted even with a 5‐year follow‐up, albeit in a reduced proportion of subjects (12%). Distinguishing by condition, in the Crohn's group, we observed fewer individuals in the low‐adherence cluster (26%) compared to the ulcerative colitis group (38%). However, in the Crohn's group, adherence levels reached 0% by the end of the 3‐year follow‐up, whereas in the ulcerative colitis group, they remained around 20%. The more severe course of CD may explain the lower population in the low‐adherence group compared to ulcerative colitis. In ulcerative colitis patients, a quicker perception of treatment efficacy might lead to a reduced perception of the need for continuous therapy.

Having used a longitudinal method to assess adherence, comparing our results with those of other studies reporting point estimates is challenging. However, one study that evaluated adherence over 2 years using a modified version of the medication possession ratio (mMPR) observed a percentage of patients meeting a specific threshold (mMPR = 100%) of 66% [11]. Another study that assessed adherence using a mMPR reported that 6.1% of patients were non‐adherent (mMPR < 90%) over 1 year of follow‐up [12]. Similarly, a study with the same follow‐up reported that 69.0% of patients who initiated the biological treatment with infliximab and 61.5% of those who initiated with vedolizumab were adherent (PDC ≥ 80%) [13]. In contrast, another study reported a lower percentage of adherent patients, with only 41% having an MPR ≥ 80% over a 1‐year follow‐up period [14]. The only study comparable to ours, as it investigated adherence longitudinally using GBTM, included a relatively small sample and focused exclusively on hospital‐based intravenous therapies [20]. As such therapies are typically administered under direct supervision, higher adherence is to be expected compared to studies like ours that include all biologics for IBD. Indeed, that study identified a larger group of consistent adherents (36.4%) than we observed (18.9%).

The main methodological difference between our study and previous research on treatment adherence to biologics for IBD lies in how the time dimension is considered in the investigation of adherence behavior. In our study, we described how adherence changed over time from treatment initiation until the end of follow‐up, whereas previous studies typically reported an overall summary measure (e.g., MPR, PDC). Furthermore, we hypothesized the existence of unobserved subgroups of individuals with similar adherence patterns, and we applied cluster analysis to identify them.

We observed that being female, having an older age, initiating biologic therapy with an originator, and taking adalimumab as the index drug are associated with low adherence. There is evidence in the literature of higher discontinuation of biologics among older patients [27].

In the literature, it has been observed that users of biologics experience an increase in depression, which may lead to a reduction in adherence to biological therapy [31].

In this regard, we observed that individuals who initiated treatment with biologics and used antidepressants in the year before the index date tended to adhere less to biologics over a period of 5 years. However, this phenomenon was not observed when considering a shorter follow‐up period (3 years). Furthermore, it is known that the prevalence of depression and anxiety disorders is higher among females [32, 33, 34, 35]. This could partly explain the observed association with sex.

In another cohort study conducted on United States users of infliximab originator and biosimilar, it was observed that among new users, adherence at 18 months was higher in subjects initiating treatment with biosimilar than with originator, despite this study being limited by a small number of biosimilar users [10].

Moreover, patients starting therapy with vedolizumab and ustekinumab reported the lowest association with low adherence with respect to those starting with adalimumab. However, by excluding patients who initiated treatment in the last three calendar years, we lost many users of vedolizumab and ustekinumab. Additionally, as for ustekinumab, the drug should be used in patients with IBDs only in case of inadequate/lost response or intolerance to cDMARDs or TNF‐alpha inhibitors, thus limiting the inclusion of incident biologic users with ustekinumab as an index drug in the study cohort (only 83 patients were included).

Furthermore, we observed that one out of three patients in the cohort (36%) had a switch of biologics within the 3‐year follow‐up period [36]. Distinguishing by condition, patients with CD switched treatments less frequently, and when they did, it occurred later than in the colitis group. Another American study, with a cohort of approximately 5000 IBD patients newly treated with biologics, reported a 3‐year switch rate of about 50%. This study included adalimumab, infliximab, golimumab, certolizumab, and vedolizumab in the analysis, making it more comparable to our study [37]. However, by excluding patients who initiated treatment in the last three calendar years, we lost many users of vedolizumab, which is a drug experiencing growth in IBD.

The median time to first switch was 1.3 years for the overall cohort over the 3‐year follow‐up period, with the lowest value of less than 1 year (346 days) observed among low adherents. It is plausible that patients who stopped biologics early had a medical switch before those who didn't, as one of the reasons for the interruptions could have been a lack of response.

Some limitations of the study warrant caution. Firstly, we do not know the reason for treatment discontinuation, and certainly, knowledge of this information influences the interpretation of observed adherence patterns. For example, we lack information on surgery, which could explain changes in adherence trends. Secondly, we limited the analysis to subjects alive up to the end of the study period. Additionally, for the estimation of therapeutic coverage, we used the DDD, which is a standard reference quantity intended to estimate drug consumption rather than coverage duration. Moreover, we considered drug dispensing as a measure of drug exposure, thus assuming for subcutaneous administration that patients ultimately took the drug as prescribed. We also did not consider the temporal variability, and specifically the indirect impact of the COVID‐19 pandemic on adherence to biologics in IBD patients. Furthermore, we may have slightly overestimated adherence, as 6% of the study cohort comprises pediatric patients, for whom the DDD is not accurate. In addition, we did not have the exact date of diagnosis, as it is not directly available in administrative databases. The date of diagnosis can be retrieved from hospital discharge records or exemptions from copayments; however, particularly for chronic conditions, these records may not accurately reflect the true date of diagnosis. Thus, a prevalent bias might be present, as our cohort might include both patients initiating biologics as first‐line treatment and those previously treated with conventional therapies. These differences in disease duration and treatment history may influence adherence to biologics in heterogeneous ways. To partially account for this, we included prior IBD treatments as covariates in the model used to predict adherence group. Nonetheless, initiation of biologics is generally considered a proxy for disease severity, as these therapies are indicated only for moderate‐to‐severe cases; therefore, all included patients can be assumed to be at a similar stage of disease severity. To the best of our knowledge, no other real‐world evidence study on adherence to biologic treatment in patients with IBD includes a cohort of this magnitude. Additionally, methods to assess biologics adherence longitudinally were used. Moreover, it is a population‐based study that includes many IBD patients from a large part of Italy, which may allow the results to be generalizable to the entire Italian population.

## 5 Conclusion

Our study highlights that one out of three patients with IBDs progressively reduced adherence to biologics within the first 3 years of use. Adherence trajectories varied according to the first biologic dispensed, its type, and patient characteristics such as sex and age, with female and older patients showing a higher likelihood of low adherence. Notably, patients with a history of antidepressant use were less likely to maintain adherence over time. This may reflect the impact of depressive symptoms, a common comorbidity among patients with IBD, that are known to negatively affect treatment adherence. These findings underscore the need for tailored adherence support strategies, particularly for women, older adults, and individuals with co‐existing depression.

## 6 Plain Language Summary

This study explored adherence behavior in a real‐world setting over a 3‐year follow‐up period to biologics in patients with inflammatory bowel diseases (IBDs) in Italy, and identified factors associated with adherence. It was a retrospective cohort study based on administrative data from 12 Italian regions within the VALORE network, corresponding to a population of almost 44 million, covering 74% of the Italian population from the North to the South and Islands. We included IBD patients with a first dispensation of biologics between 2010 and 2019. Monthly adherence was computed, and statistical methods based on cluster analysis were used to identify adherence groups. We identified three patterns of adherence: one included patients who remained adherent to biologics; another included those who initially reduced adherence but stabilized at about 70% after a few months of use, and the last one comprised individuals who gradually decreased their adherence over time. Women, older patients, and those starting with adalimumab were more likely to exhibit low adherence, while those who began with a biosimilar were less likely to have low adherence. These findings highlight that adherence to biologics presents differences based on patient characteristics and the type of biologic used. Understanding these patterns can help healthcare providers identify at‐risk groups and tailor strategies to improve long‐term treatment adherence.

## Author Contributions

Conceptualization: Sabrina Giometto, Andrea Spini, Ersilia Lucenteforte, Gianluca Trifirò. Data curation: Giorgia Pellegrini, Andrea Spini, Luca L'Abbate. Formal analysis: Sabrina Giometto, Giorgia Pellegrini, Ersilia Lucenteforte. Funding acquisition: Gianluca Trifirò. Methodology: Sabrina Giometto, Andrea Spini, Ersilia Lucenteforte, Gianluca Trifirò. Project administration: Gianluca Trifirò. Supervision: Gianluca Trifirò, Ersilia Lucenteforte. Writing – original draft: Sabrina Giometto. Writing – review and editing: all authors.

## Conflicts of Interest

Ylenia Ingrasciotta is the CEO of the academic spin‐off “INSPIRE srl,” which has received funding for conducting observational studies from contract research organizations (RTI Health Solutions, Pharmo Institute N.V.) and from pharmaceutical companies (Chiesi Italia, Kyowa Kirin s.r.l., Daiichi Sankyo Italia S.p.A.). Gianluca Trifirò participated in advisory boards and seminars as a lecturer on topics not related to the paper and sponsored by the following pharmaceutical companies in the last 2 years: Eli Lilly, Sanofi, Amgen, Novo Nordisk, Sobi, Gilead, Celgene, Daiichi Sankyo, Takeda, and MSD. He is also the scientific coordinator of the pharmacoepidemiology team at the University of Verona and of the academic spin‐off “INSPIRE srl” that carried out in the last 2 years observational studies/systematic reviews on topics not related to the content of this article and which were funded by PTC Pharmaceutics, Kyowa Kirin, Shionogi, Shire, Chiesi, and Daiichi Sankyo. Ersilia Lucenteforte was involved as an investigator of observational studies funded by the pharmaceutical company Galapagos in compliance with the ENCEPP Code of Conduct, and she has carried out consultancy for Angelini.

## Supporting information


**Table S1:** Defined daily doses assigned by the WHO.
**Table S2:** Codes used to identify comorbidities.
**Table S3:** Codes used to identify drug therapies.
**Table S4:** Distribution of cohort characteristics at index date and 1 year before, by index drug, for a cohort with at least a 3‐year follow‐up period.
**Table S5:** Distribution of cohort characteristics at index date and 1 year before, by cluster for a cohort with at least a 3‐year follow‐up period.
**Table S6:** Distribution of cohort characteristics at index date and 1 year before for Crohn's disease only, by cluster for a cohort with at least a 3‐year follow‐up period.
**Table S7:** Distribution of cohort characteristics at index date and 1 year before for ulcerative colitis only, by cluster for a cohort with at least a 3‐year follow‐up period.
**Table S8:** Distribution of cohort characteristics at index date and 1 year before, overall and by cluster for a cohort with at least a 5‐year follow‐up period.
**Table S9:** Distribution of non‐biological drugs and biological drugs (switch) users during a 5‐year follow‐up period, overall and by cluster for a cohort with at least a 5‐year follow‐up period.
**Figure S1:** Study design implementation.
**Figure S2:** Trajectories of adherence to biologics for IBDs for a cohort with at least a 5‐year follow‐up period.
**Figure S3:** Determinants of treatment adherence to biologics for a cohort with at least a 5‐year follow‐up period.

## Data Availability

Individual research data cannot be shared due to privacy issues.
