# Supplementary material for: Trajectories of Adherence to Biologics in Patients With Inflammatory Bowel Diseases: A Large‐Scale Multi‐Regional Italian Study Through the VALORE Distributed Database
Source: Pharmacoepidemiol Drug Saf. 2025 Oct 9;34(10):e70230. doi: 10.1002/pds.70230 (PMC12511659; doi:10.1002/pds.70230)

**Table S1.** Defined daily doses assigned by the WHO

| **AIC** | **Name** | **Route of administration** | **ATC** | **DDD** | **Days covered** |
| --- | --- | --- | --- | --- | --- |
| 045317017 | Adalimumab | Parenteral | L04AB04 | 2.9 mg | 6.9 |
| 047805015 |  |  |  |  | 13.8 |
| 047088087 |  |  |  |  | 13.8 |
| 047088048 |  |  |  |  | 13.8 |
| 047088012 |  |  |  |  | 13.8 |
| 046889073 |  |  |  |  | 13.8 |
| 046889046 |  |  |  |  | 13.8 |
| 046889010 |  |  |  |  | 13.8 |
| 046888071 |  |  |  |  | 13.8 |
| 046888044 |  |  |  |  | 13.8 |
| 046888018 |  |  |  |  | 13.8 |
| 046887042 |  |  |  |  | 13.8 |
| 046887016 |  |  |  |  | 13.8 |
| 045616051 |  |  |  |  | 13.8 |
| 045616012 |  |  |  |  | 13.8 |
| 045317068 |  |  |  |  | 13.8 |
| 045317029 |  |  |  |  | 13.8 |
| 035946223 |  |  |  |  | 13.8 |
| 035946159 |  |  |  |  | 13.8 |
| 035946110 |  |  |  |  | 13.8 |
| 035946060 |  |  |  |  | 13.8 |
| 035946211 |  |  |  |  | 13.8 |
| 035946072 |  |  |  |  | 13.8 |
| 035946021 |  |  |  |  | 13.8 |
| 049376015 |  |  |  |  | 13.8 |
| 049376054 |  |  |  |  | 13.8 |
| 049376092 |  |  |  |  | 13.8 |
| 047805039 |  |  |  |  | 27.6 |
| 047805027 |  |  |  |  | 27.6 |
| 047088075 |  |  |  |  | 27.6 |
| 047088051 |  |  |  |  | 27.6 |
| 047088024 |  |  |  |  | 27.6 |
| 046889059 |  |  |  |  | 27.6 |
| 046889022 |  |  |  |  | 27.6 |
| 046888057 |  |  |  |  | 27.6 |
| 046888020 |  |  |  |  | 27.6 |
| 046887055 |  |  |  |  | 27.6 |
| 046887028 |  |  |  |  | 27.6 |
| 045616099 |  |  |  |  | 27.6 |
| 035946250 |  |  |  |  | 27.6 |
| 035946235 |  |  |  |  | 27.6 |
| 045616063 |  |  |  |  | 27.6 |
| 045616024 |  |  |  |  | 27.6 |
| 045317070 |  |  |  |  | 27.6 |
| 045317031 |  |  |  |  | 27.6 |
| 035946197 |  |  |  |  | 27.6 |
| 035946161 |  |  |  |  | 27.6 |
| 035946122 |  |  |  |  | 27.6 |
| 035946084 |  |  |  |  | 27.6 |
| 035946033 |  |  |  |  | 27.6 |
| 035946019 |  |  |  |  | 27.6 |
| 049376027 |  |  |  |  | 27.6 |
| 049376066 |  |  |  |  | 27.6 |
| 049376104 |  |  |  |  | 27.6 |
| 049376130 |  |  |  |  | 27.6 |
| 049376142 |  |  |  |  | 27.6 |
| 049376155 |  |  |  |  | 27.6 |
| 049767027 |  |  |  |  | 27.6 |
| 049767054 |  |  |  |  | 27.6 |
| 049767078 |  |  |  |  | 27.6 |
| 035946209 |  |  |  |  | 27.6 |
| 045616075 |  |  |  |  | 55.2 |
| 045616036 |  |  |  |  | 55.2 |
| 045317082 |  |  |  |  | 55.2 |
| 045317043 |  |  |  |  | 55.2 |
| 035946173 |  |  |  |  | 55.2 |
| 035946096 |  |  |  |  | 55.2 |
| 035946045 |  |  |  |  | 55.2 |
| 049376039 |  |  |  |  | 55.2 |
| 049376116 |  |  |  |  | 55.2 |
| 049376078 |  |  |  |  | 55.2 |
| 035946134 |  |  |  |  | 55.2 |
| 047088063 |  |  |  |  | 82.8 |
| 047088036 |  |  |  |  | 82.8 |
| 046889061 |  |  |  |  | 82.8 |
| 046889034 |  |  |  |  | 82.8 |
| 046888069 |  |  |  |  | 82.8 |
| 046888032 |  |  |  |  | 82.8 |
| 046887067 |  |  |  |  | 82.8 |
| 046887030 |  |  |  |  | 82.8 |
| 045616087 |  |  |  |  | 82.8 |
| 045616048 |  |  |  |  | 82.8 |
| 045317094 |  |  |  |  | 82.8 |
| 045317056 |  |  |  |  | 82.8 |
| 035946262 |  |  |  |  | 82.8 |
| 035946247 |  |  |  |  | 82.8 |
| 035946185 |  |  |  |  | 82.8 |
| 035946146 |  |  |  |  | 82.8 |
| 035946108 |  |  |  |  | 82.8 |
| 035946058 |  |  |  |  | 82.8 |
| 049376041 |  |  |  |  | 82.8 |
| 049376080 |  |  |  |  | 82.8 |
| 049376128 |  |  |  |  | 82.8 |
| 049376167 |  |  |  |  | 82.8 |
| 047805054 |  |  |  |  | 82.8 |
| 047805041 |  |  |  |  | 82.8 |
| 039541091 | Golimumab | Parenteral | L04AB06 | 1.66 mg | 27.1 |
| 039541014 |  |  |  |  | 30.1 |
| 039541038 |  |  |  |  | 30.1 |
| 039541053 |  |  |  |  | 60.2 |
| 039541077 |  |  |  |  | 60.2 |
| 039541026 |  |  |  |  | 90.4 |
| 039541040 |  |  |  |  | 90.4 |
| 039541065 |  |  |  |  | 180.7 |
| 039541089 |  |  |  |  | 180.7 |
| 046635013 | Infliximab | Parenteral | L04AB02 | 3.75 mg | 26.7 |
| 044892014 |  |  |  |  | 26.7 |
| 043010014 |  |  |  |  | 26.7 |
| 042942019 |  |  |  |  | 26.7 |
| 034528012 |  |  |  |  | 26.7 |
| 042942122 |  |  |  |  | 32.0 |
| 042942096 |  |  |  |  | 32.0 |
| 042942060 |  |  |  |  | 32.0 |
| 046635025 |  |  |  |  | 53.3 |
| 044892026 |  |  |  |  | 53.3 |
| 042942021 |  |  |  |  | 53.3 |
| 043010026 |  |  |  |  | 53.3 |
| 042942134 |  |  |  |  | 64.0 |
| 042942108 |  |  |  |  | 64.0 |
| 042942072 |  |  |  |  | 64.0 |
| 046635037 |  |  |  |  | 80.0 |
| 044892038 |  |  |  |  | 80.0 |
| 043010038 |  |  |  |  | 80.0 |
| 042942033 |  |  |  |  | 80.0 |
| 046635049 |  |  |  |  | 106.7 |
| 044892040 |  |  |  |  | 106.7 |
| 042942045 |  |  |  |  | 106.7 |
| 043010040 |  |  |  |  | 106.7 |
| 042942146 |  |  |  |  | 128.0 |
| 042942110 |  |  |  |  | 128.0 |
| 042942084 |  |  |  |  | 128.0 |
| 046635052 |  |  |  |  | 133.3 |
| 044892053 |  |  |  |  | 133.3 |
| 042942058 |  |  |  |  | 133.3 |
| 043010053 |  |  |  |  | 133.3 |
| 038936035 | Ustekinumab | Parenteral | L04AC05 | 0.54 mg | 83.3 |
| 038936011 |  |  |  |  | 83.3 |
| 038936047 |  |  |  |  | 166.7 |
| 038936023 |  |  |  |  | 166.7 |
| 038936050 |  |  |  |  | 240.7 |
| 043442058 | Vedolizumab | Parenteral | L04AA33 | 5.4 mg | 20.0 |
| 043442021 |  |  |  |  | 20.0 |
| 043442060 |  |  |  |  | 40.0 |
| 043442033 |  |  |  |  | 40.0 |
| 043442019 |  |  |  |  | 55.6 |
| 043442072 |  |  |  |  | 120.0 |
| 043442045 |  |  |  |  | 120.0 |

AIC: marketing authorisation code; ATC: anatomical therapeutic chemical; DDD: defined daily dose

**Table S2.** Codes used to identify comorbidities

|  | **ICD-9-CM** | **Exemption codes** |
| --- | --- | --- |
| Hypertension | 401-405 | 031 |
| Atrial fibrillation | 4273 | - |
| Ischemic heart disease | 410,411,413,414,4260, 4275,V450,V4581,V4582 | - |
| Cerebrovascular disease | 430-438 | - |
| Chronic pulmonary disease | 491 | 057 |
| Diabetes mellitus | 249,250,3572,3620,36641,6488,7751,79029 | 013,250 |
| Liver disease | 570-573,57142 | 016 |
| Neoplasms | 140-209,230-239,1623-1625,1628,1629,1874,1877,2028, 2041,2302, 2330, 2393,20931,V10 | 048 |
| Other immune-mediated inflammatory diseases (IMIDs) | 36011,36012,36320,364,6960,6961,70583,714,720 | 006,028,030,0456960,0456961,054,6960,6961,7100,714,7200 |
| Chronic kidney disease failure | 584-586 | 023 |

ICD-9-CM: International Classification of Diseases, 9th Revision, Clinical Modification

**Table S3.** Codes used to identify drug therapies

|  | **ATC codes** |
| --- | --- |
| Antiplatelet drugs | B01AC |
| Antiarrhythmics | C01B |
| Antibacterial for systemic use | J01 |
| Anticoagulants | B01AB |
| Antidepressants | N06A |
| Antipsychotics | N05A |
| Antivirals for systemic use | J05 |
| Conventional synthetic DMARDs | A07EC01, L01BA01, L04AX03, A07EC02, A07EC04 |
| Drugs for peptic ulcer and gastro-esophageal reflux disease | A02B |
| Glucocorticoids for systemic use | H02AB, R03BA |
| Non-steroidal anti-inflammatory drugs (NSAIDs) | M01A |

ATC: Anatomical Therapeutic Chemical; DMARDs: disease-modifying antirheumatic drugs

**Table S4.** Distribution of cohort characteristics at index date and one year before, by index drug, for a cohort with at least a 3-year follow-up period

|  | **Adalimumab**  **(N=9,540)** | **Golimumab (N=991)** | **Infliximab (N=8,307)** | **Ustekinumab (N=83)** | **Vedolizumab (N=1,229)** |
| --- | --- | --- | --- | --- | --- |
| **At index date** | | | | | |
| Sex, males, N (%) | 5,084 (53.3) | 545 (55.0) | 4,764 (57.3) | 47 (56.6) | 744 (60.5) |
| Age, N (%) |  |  |  |  |  |
| < 18 | 531 (5.6) | - | 695 (8.4) | 1 (1.2) | 7 (0.6) |
| 18-44 | 5,067 (53.1) | 467 (47.1) | 4146 (49.9) | 35 (42.2) | 310 (25.2) |
| 45-64 | 3,421 (35.9) | 434 (43.8) | 2,958 (35.6) | 26 (31.3) | 419 (34.1) |
| 65-79 | 510 (5.3) | 89 (9.0) | 500 (6.0) | 18 (21.7) | 451 (36.7) |
| ≥80 | 11 (0.1) | 1 (0.1) | 8 (0.1) | 3 (3.6) | 42 (3.4) |
| Indication of use, N (%) |  |  |  |  |  |
| Crohn | 7,013 (73.5) | 154 (15.5) | 3,981 (47.9) | 81 (97.6) | 491 (40.0) |
| Ulcerative colitis | 2,527 (26.5) | 837 (84.5) | 4,326 (52.1) | 2 (2.4) | 738 (60.0) |
| Type of index drug, N (%) |  |  |  |  |  |
| Biosimilar | 800 (8.4) | - | 3,707 (44.6) | - | - |
| Originator | 8,740 (91.6) | 991 (100.0) | 4,600 (55.4) | 83 (100.0) | 1,229 (100.0) |
| **Before index date (ever)** | | | | | |
| History of comorbidity, N (%) | | | | | |
| Hypertension | 712 (7.5) | 109 (11.0) | 692 (8.3) | 16 (19.3) | 274 (22.3) |
| Atrial fibrillation | 43 (0.5) | 5 (0.5) | 52 (0.6) | 1 (1.2) | 60 (4.9) |
| Ischemic hearth disease | 124 (1.3) | 14 (1.4) | 109 (1.3) | 5 (6.0) | 88 (7.2) |
| Cerebrovascular disease | 93 (1.0) | 17 (1.7) | 85 (1.0) | 4 (4.8) | 31 (2.5) |
| Diabetes mellitus | 296 (3.1) | 45 (4.5) | 372 (4.5) | 5 (6.0) | 112 (9.1) |
| Chronic renal disease | 73 (0.8) | 10 (1.0) | 68 (0.8) | 2 (2.4) | 42 (3.4) |
| Chronic liver disease | 75 (0.8) | 13 (1.3) | 87 (1.0) | 3 (3.6) | 26 (2.1) |
| Chronic pulmonary disease | 76 (0.8) | 7 (0.7) | 72 (0.9) | 4 (4.8) | 33 (2.7) |
| History of tumors | 346 (3.6) | 41 (4.1) | 324 (3.9) | 9 (10.8) | 217 (17.7) |
| Intestinal infections | 1,159 (12.1) | 53 (5.3) | 956 (11.5) | 16 (19.3) | 129 (10.5) |
| Other IMIDs |  |  |  |  |  |
| Psoriasis | 486 (5.1) | 46 (4.6) | 306 (3.7) | 25 (30.1) | 71 (5.8) |
| Psoriatic arthritis | 351 (3.7) | 55 (5.5) | 170 (2.0) | 11 (13.3) | 34 (2.8) |
| Rheumatoid arthritis | 305 (3.2) | 37 (3.7) | 146 (1.8) | 3 (3.6) | 24 (2.0) |
| Ankylosing spondylitis | 566 (5.9) | 58 (5.9) | 250 (3.0) | 3 (3.6) | 19 (1.5) |
| **Before index date (one year)** | | | | | |
| Previous use of drugs, N (%) | | | | | |
| Antiplatelet agents | 29 (0.3) | 2 (0.2) | 30 (0.4) | 1 (1.2) | 5 (0.4) |
| Antiarrhythmics | 7 (0.1) | - | 6 (0.1) | - | 2 (0.2) |
| Anticoagulants | 392 (4.1) | 18 (1.8) | 240 (2.9) | 5 (6.0) | 37 (3.0) |
| Antidepressants | 71 (0.7) | 11 (1.1) | 90 (1.1) | - | 16 (1.3) |
| Antipsychotics | 20 (0.2) | 1 (0.1) | 19 (0.2) | 1 (1.2) | 4 (0.3) |
| Antibacterial for systemic use | 44 (0.5) | 6 (0.6) | 55 (0.7) | - | 6 (0.5) |
| Antivirals for systemic use | 90 (0.9) | 18 (1.8) | 108 (1.3) | 2 (2.4) | 23 (1.9) |
| Drugs for peptic ulcer and gastro-oesophageal reflux disease | 301 (3.2) | 28 (2.8) | 288 (3.5) | 1 (1.2) | 18 (1.5) |
| csDMARDs | 706 (7.4) | 38 (3.8) | 571 (6.9) | 9 (10.8) | 72 (5.9) |
| Methotrexate | 113 (1.2) | 6 (0.6) | 55 (0.7) | 3 (3.6) | 13 (1.1) |
| Sulfasalazine | 132 (1.4) | 16 (1.6) | 97 (1.2) | 2 (2.4) | 9 (0.7) |
| Mesalazine | 478 (5.0) | 21 (2.1) | 421 (5.1) | 4 (4.8) | 51 (4.1) |
| Balsalazide | 7 (0.1) | - | 10 (0.1) | - | - |
| Glucocorticoids | 200 (2.1) | 10 (1.0) | 153 (1.8) | 4 (4.8) | 22 (1.8) |
| NSAIDs | 161 (1.7) | 16 (1.6) | 134 (1.6) | 1 (1.2) | 14 (1.1) |

csDMARDs: conventional synthetic disease-modifying antirheumatic drugs; NSAIDs: nonsteroidal anti-inflammatory drugs

**Table S5.** Distribution of cohort characteristics at index date and one year before, by cluster for a cohort with at least a 3-year follow-up period

|  | **High adherence**  **(N=3,799)** | **Medium adherence**  **(N=9,351)** | **Low adherence**  **(N=7,000)** |
| --- | --- | --- | --- |
| **At index date** | | | |
| Sex, males, N (%) | 2,424 (63.8) | 4,994 (53.4) | 3,766 (53.8) |
| Age,N (%) |  |  |  |
| < 18 | 234 (6.2) | 629 (6.7) | 371 (5.3) |
| 18-44 | 1,826 (48.1) | 4,738 (50.7) | 3,461 (49.4) |
| 45-64 | 1,428 (37.6) | 3,303 (35.3) | 2,527 (36.1) |
| 65-79 | 294 (7.7) | 660 (7.1) | 614 (8.8) |
| ≥80 | 17 (0.4) | 21 (0.2) | 27 (0.4) |
| Indication of use, N (%) |  |  |  |
| Crohn | 1,942 (51.1) | 6,128 (65.5) | 3,650 (52.1) |
| Ulcerative colitis | 1,857 (48.9) | 3,223 (34.5) | 3,350 (47.9) |
| Index drug, N (%) |  |  |  |
| Adalimumab | 1,008 (26.5) | 5,840 (62.5) | 2,692 (38.5) |
| Golimumab | 217 (5.7) | 455 (4.9) | 319 (4.6) |
| Infliximab | 2,203 (58.0) | 2,462 (26.3) | 3,642 (52.0) |
| Ustekinumab | 51 (1.3) | 15 (0.2) | 17 (0.2) |
| Vedolizumab | 320 (8.4) | 579 (6.2) | 330 (4.7) |
| Index route of administration, N (%) |  |  |  |
| Intravenous | 2,554 (67.2) | 3,045 (32.6) | 3,976 (56.8) |
| Subcutaneous | 1,245 (32.8) | 6,306 (67.4) | 3,024 (43.2) |
| Index class, N (%) |  |  |  |
| Interleukins | 51 (1.3) | 15 (0.2) | 17 (0.2) |
| Selective immunosuppressant | 320 (8.4) | 579 (6.2) | 330 (4.7) |
| Tumor necrosis factor | 3,428 (90.2) | 8,757 (93.6) | 6,653 (95.0) |
| Type of index drug, N (%) |  |  |  |
| Biosimilar | 1,338 (35.2) | 1,625 (17.4) | 1,544 (22.1) |
| Originator | 2,461 (64.8) | 7,726 (82.6) | 5,456 (77.9) |
| **Before index date (ever)** | | | |
| History of comorbidity, N (%) | | | |
| Hypertension | 355 (9.3) | 786 (8.4) | 662 (9.5) |
| Atrial fibrillation | 31 (0.8) | 72 (0.8) | 58 (0.8) |
| Ischemic hearth disease | 69 (1.8) | 139 (1.5) | 132 (1.9) |
| Cerebrovascular disease | 34 (0.9) | 115 (1.2) | 81 (1.2) |
| Diabetes mellitus | 160 (4.2) | 326 (3.5) | 344 (4.9) |
| Chronic renal disease | 32 (0.8) | 88 (0.9) | 75 (1.1) |
| Chronic liver disease | 36 (0.9) | 87 (0.9) | 81 (1.2) |
| Chronic pulmonary disease | 48 (1.3) | 77 (0.8) | 67 (1.0) |
| History of tumors | 174 (4.6) | 420 (4.5) | 343 (4.9) |
| Intestinal infections | 396 (10.4) | 1,122 (12.0) | 795 (11.4) |
| Other IMIDs |  |  |  |
| Psoriasis | 164 (4.3) | 459 (4.9) | 311 (4.4) |
| Psoriatic arthritis | 102 (2.7) | 322 (3.4) | 197 (2.8) |
| Rheumatoid arthritis | 89 (2.3) | 264 (2.8) | 162 (2.3) |
| Ankylosing spondylitis | 128 (3.4) | 523 (5.6) | 245 (3.5) |
| **Before index date (one year)** | | | |
| Previous use of drugs, N (%) | | | |
| Antiplatelet agents | 10 (0.3) | 30 (0.3) | 27 (0.4) |
| Antiarrhythmics | 1 (0.0) | 8 (0.1) | 6 (0.1) |
| Anticoagulants | 146 (3.8) | 357 (3.8) | 189 (2.7) |
| Antidepressants | 37 (1.0) | 90 (1.0) | 61 (0.9) |
| Antipsychotics | 11 (0.3) | 20 (0.2) | 14 (0.2) |
| Antibacterial for systemic use | 32 (0.8) | 49 (0.5) | 30 (0.4) |
| Antivirals for systemic use | 50 (1.3) | 113 (1.2) | 78 (1.1) |
| Drugs for peptic ulcer and gastro-oesophageal reflux disease | 139 (3.7) | 318 (3.4) | 179 (2.6) |
| csDMARDs | 279 (7.3) | 652 (7.0) | 465 (6.6) |
| Methotrexate | 37 (1.0) | 81 (0.9) | 72 (1.0) |
| Sulfasalazine | 43 (1.1) | 128 (1.4) | 85 (1.2) |
| Mesalazine | 206 (5.4) | 451 (4.8) | 318 (4.5) |
| Balsalazide | 4 (0.1) | 6 (0.1) | 7 (0.1) |
| Glucocorticoids | 80 (2.1) | 189 (2.0) | 120 (1.7) |
| NSAIDs | 61 (1.6) | 164 (1.8) | 101 (1.4) |

csDMARDs: conventional synthetic disease-modifying antirheumatic drugs; NSAIDs: nonsteroidal anti-inflammatory drugs

**Table S6.** Distribution of cohort characteristics at index date and one year before for Crohn disease only, by cluster for a cohort with at least a 3-year follow-up period

|  | **Overall**  (N = 11,720) | **High adherence**  (N = 4,361) | **Medium adherence**  (N = 4,338) | **Low adherence**  (N = 3,021) |
| --- | --- | --- | --- | --- |
| **At index date** |  |  |  |  |
| Sex, males, N (%) | 6,422 (54.8) | 2,672 (61.3) | 2,253 (51.9) | 1,497 (49.6) |
| Age, >65, N (%) | 710 (6.1) | 276 (6.3) | 234 (5.4) | 200 (6.6) |
| Index drug, N (%) |  |  |  |  |
| Adalimumab | 7,013 (59.8) | 1,922 (44.1) | 3,361 (77.5) | 1,730 (57.3) |
| Golimumab | 154 (1.3) | 51 (1.2) | 68 (1.6) | 35 (1.2) |
| Infliximab | 3,981 (34.0) | 2,076 (47.6) | 757 (17.5) | 1,148 (38.0) |
| Ustekinumab | 81 (0.7) | 63 (1.4) | 5 (0.1) | 13 (0.4) |
| Vedolizumab | 491 (4.2) | 249 (5.7) | 147 (3.4) | 95 (3.1) |
| Index route of administration, N (%) |  |  |  |  |
| Intravenous | 7,210 (61.5) | 2,001 (45.9) | 3,433 (79.1) | 1,776 (58.8) |
| Subcutaneous | 4,510 (38.5) | 2,360 (54.1) | 905 (20.9) | 1,245 (41.2) |
| Index class, N (%) |  |  |  |  |
| Interleukin | 81 (0.7) | 63 (1.4) | 5 (0.1) | 13 (0.4) |
| Selective immunosuppressant | 491 (4.2) | 249 (5.7) | 147 (3.4) | 95 (3.1) |
| Tumor necrosis factor | 11,148 (95.1) | 4,049 (92.8) | 4,186 (96.5) | 2,913 (96.4) |
| Type of index drug, N (%) |  |  |  |  |
| Originator | 9,434 (80.5) | 3,110 (71.3) | 3,783 (87.2) | 2,541 (84.1) |
| Biosimilar | 2,286 (19.5) | 1,251 (28.7) | 555 (12.8) | 480 (15.9) |
| **Before index date (ever)** |  |  |  |  |
| History of comorbidity, N (%) |  |  |  |  |
| Hypertension | 898 (7.7) | 308 (7.1) | 344 (7.9) | 246 (8.1) |
| Atrial fibrillation | 77 (0.7) | 35 (0.8) | 23 (0.5) | 19 (0.6) |
| Ischemic heart disease | 150 (1.3) | 51 (1.2) | 50 (1.2) | 49 (1.6) |
| Cerebrovascular disease | 121 (1.0) | 51 (1.2) | 42 (1.0) | 28 (0.9) |
| Diabete mellitus | 356 (3.0) | 134 (3.1) | 117 (2.7) | 105 (3.5) |
| Chronic renal disease | 110 (0.9) | 34 (0.8) | 39 (0.9) | 37 (1.2) |
| Chronic liver disease | 104 (0.9) | 41 (0.9) | 32 (0.7) | 31 (1.0) |
| Chronic pulmonary disease | 115 (1.0) | 48 (1.1) | 36 (0.8) | 31 (1.0) |
| History of tumors | 520 (4.4) | 200 (4.6) | 188 (4.3) | 132 (4.4) |
| Intestinal infections | 1,690 (14.4) | 621 (14.2) | 630 (14.5) | 439 (14.5) |
| Psoriasis | 540 (4.6) | 191 (4.4) | 214 (4.9) | 135 (4.5) |
| Psoriatic arthritis | 354 (3.0) | 115 (2.6) | 151 (3.5) | 88 (2.9) |
| Reumatoid arthritis | 281 (2.4) | 98 (2.2) | 113 (2.6) | 70 (2.3) |
| Ankylosing spondylitis | 546 (4.7) | 173 (4.0) | 258 (5.9) | 115 (3.8) |
| **Before index date (one year)** |  |  |  |  |
| Previous use of drugs, N (%) |  |  |  |  |
| Antiplatelet agents | 40 (0.3) | 14 (0.3) | 16 (0.4) | 10 (0.3) |
| Antiarrhythmics | 10 (0.1) | 4 (0.1) | 1 (0.0) | 5 (0.2) |
| Anticoagulants | 522 (4.5) | 204 (4.7) | 212 (4.9) | 106 (3.5) |
| Antidepressants | 89 (0.8) | 31 (0.7) | 30 (0.7) | 28 (0.9) |
| Antipsychotics | 25 (0.2) | 12 (0.3) | 7 (0.2) | 6 (0.2) |
| Antibacterial for systemic use | 68 (0.6) | 32 (0.7) | 20 (0.5) | 16 (0.5) |
| Antivirals for systemic use | 123 (1.0) | 50 (1.1) | 52 (1.2) | 21 (0.7) |
| Drugs for peptic ulcer and gastro-oesophageal reflux disease | 353 (3.0) | 140 (3.2) | 143 (3.3) | 70 (2.3) |
| csDMARDs | 896 (7.6) | 373 (8.6) | 313 (7.2) | 210 (7.0) |
| Methotrexate | 139 (1.2) | 47 (1.1) | 47 (1.1) | 45 (1.5) |
| Sulfasalazine | 146 (1.2) | 56 (1.3) | 56 (1.3) | 34 (1.1) |
| Mesalazine | 633 (5.4) | 279 (6.4) | 215 (5.0) | 139 (4.6) |
| Balsalazide | 5 (0.0) | 2 (0.0) | 2 (0.0) | 1 (0.0) |
| Glucocorticoids | 255 (2.2) | 93 (2.1) | 89 (2.1) | 73 (2.4) |
| NSAIDs | 177 (1.5) | 58 (1.3) | 73 (1.7) | 46 (1.5) |

csDMARDs: conventional synthetic disease-modifying antirheumatic drugs; NSAIDs: nonsteroidal anti-inflammatory drugs

**Table S7.** Distribution of cohort characteristics at index date and one year before for Ulcerative colitis only, by cluster for a cohort with at least a 3-year follow-up period

|  | **Overall**  (N = 8,430) | **High adherence**  (N = 1,853) | **Medium adherence**  (N = 3,393) | **Low adherence**  (N = 3,184) |
| --- | --- | --- | --- | --- |
| **At index date** |  |  |  |  |
| Sex, males, N (%) | 4,762 (56.5) | 1,185 (64.0) | 1,787 (52.7) | 1,790 (56.2) |
| Age, >65, N (%) | 699 (8.3) | 141 (7.6) | 261 (7.7) | 297 (9.3) |
| Index drug, N (%) |  |  |  |  |
| Adalimumab | 2,527 (30.0) | 354 (19.1) | 1,427 (42.1) | 746 (23.4) |
| Golimumab | 837 (9.9) | 199 (10.7) | 381 (11.2) | 257 (8.1) |
| Infliximab | 4,326 (51.3) | 1,116 (60.2) | 1,222 (36.0) | 1,988 (62.4) |
| Ustekinumab | 2 (0.0) | 1 (0.1) | 0 (0.0) | 1 (0.0) |
| Vedolizumab | 738 (8.8) | 183 (9.9) | 363 (10.7) | 192 (6.0) |
| Index route of administration, N (%) |  |  |  |  |
| Intravenous | 3,365 (39.9) | 554 (29.9) | 1,808 (53.3) | 1,003 (31.5) |
| Subcutaneous | 5,065 (60.1) | 1,299 (70.1) | 1,585 (46.7) | 2,181 (68.5) |
| Index class, N (%) |  |  |  |  |
| Interleukin | 2 (0.0) | 1 (0.1) | 0 (0.0) | 1 (0.0) |
| Selective immunosuppressant | 738 (8.8) | 183 (9.9) | 363 (10.7) | 192 (6.0) |
| Tumor necrosis factor | 7,690 (91.2) | 1,669 (90.1) | 3,030 (89.3) | 2,991 (93.9) |
| Type of index drug, N (%) |  |  |  |  |
| Originator | 6,209 (73.7) | 1,187 (64.1) | 2,631 (77.5) | 2,391 (75.1) |
| Biosimilar | 2,221 (26.3) | 666 (35.9) | 762 (22.5) | 793 (24.9) |
| **Before index date (ever)** |  |  |  |  |
| History of comorbidity, N (%) |  |  |  |  |
| Hypertension | 905 (10.7) | 206 (11.1) | 342 (10.1) | 357 (11.2) |
| Atrial fibrillation | 84 (1.0) | 13 (0.7) | 37 (1.1) | 34 (1.1) |
| Ischemic heart disease | 190 (2.3) | 45 (2.4) | 75 (2.2) | 70 (2.2) |
| Cerebrovascular disease | 109 (1.3) | 19 (1.0) | 48 (1.4) | 42 (1.3) |
| Diabete mellitus | 474 (5.6) | 86 (4.6) | 178 (5.2) | 210 (6.6) |
| Chronic renal disease | 85 (1.0) | 17 (0.9) | 33 (1.0) | 35 (1.1) |
| Chronic liver disease | 100 (1.2) | 20 (1.1) | 37 (1.1) | 43 (1.4) |
| Chronic pulmonary disease | 77 (0.9) | 20 (1.1) | 26 (0.8) | 31 (1.0) |
| History of tumors | 417 (4.9) | 78 (4.2) | 163 (4.8) | 176 (5.5) |
| Intestinal infections | 623 (7.4) | 117 (6.3) | 248 (7.3) | 258 (8.1) |
| Psoriasis | 394 (4.7) | 69 (3.7) | 188 (5.5) | 137 (4.3) |
| Psoriatic arthritis | 267 (3.2) | 44 (2.4) | 132 (3.9) | 91 (2.9) |
| Reumatoid arthritis | 234 (2.8) | 41 (2.2) | 124 (3.7) | 69 (2.2) |
| Ankylosing spondylitis | 350 (4.2) | 50 (2.7) | 200 (5.9) | 100 (3.1) |
| **Before index date (one year)** | | | | |
| Previous use of drugs, N (%) | | | | |
| Antiplatelet agents | 27 (0.3) | 4 (0.2) | 9 (0.3) | 14 (0.4) |
| Antiarrhythmics | 5 (0.1) | 0 (0.0) | 2 (0.1) | 3 (0.1) |
| Anticoagulants | 170 (2.0) | 31 (1.7) | 75 (2.2) | 64 (2.0) |
| Antidepressants | 99 (1.2) | 20 (1.1) | 46 (1.4) | 33 (1.0) |
| Antipsychotics | 20 (0.2) | 4 (0.2) | 10 (0.3) | 6 (0.2) |
| Antibacterial for systemic use | 43 (0.5) | 14 (0.8) | 17 (0.5) | 12 (0.4) |
| Antivirals for systemic use | 118 (1.4) | 25 (1.3) | 41 (1.2) | 52 (1.6) |
| Drugs for peptic ulcer and gastro-oesophageal reflux disease | 283 (3.4) | 69 (3.7) | 133 (3.9) | 81 (2.5) |
| csDMARDs | 500 (5.9) | 117 (6.3) | 193 (5.7) | 190 (6.0) |
| Methotrexate | 51 (0.6) | 14 (0.8) | 19 (0.6) | 18 (0.6) |
| Sulfasalazine | 110 (1.3) | 17 (0.9) | 46 (1.4) | 47 (1.5) |
| Mesalazine | 342 (4.1) | 87 (4.7) | 129 (3.8) | 126 (4.0) |
| Balsalazide | 12 (0.1) | 3 (0.2) | 3 (0.1) | 6 (0.2) |
| Glucocorticoids | 134 (1.6) | 36 (1.9) | 61 (1.8) | 37 (1.2) |
| NSAIDs | 149 (1.8) | 34 (1.8) | 72 (2.1) | 43 (1.4) |

csDMARDs: conventional synthetic disease-modifying antirheumatic drugs; NSAIDs: nonsteroidal anti-inflammatory drugs

**Table S8**. Distribution of cohort characteristics at index date and one year before, overall and by cluster for a cohort with at least a 5-year follow-up period

|  | **Overall**  **(N= 13,041)** | **High adherence**  **(N = 1,525)** | **Medium adherence (N=6,810)** | **Low adherence (N=4,706)** |
| --- | --- | --- | --- | --- |
| **At index date** | | | | |
| Sex, males, N (%) | 7,138 (54.7) | 1,012 (66.4) | 3,691 (54.2) | 2,435 (51.7) |
| Age,≥65, N (%) | 705 ( 5.4) | 72 (4.7) | 297 (4.4) | 336 (7.1) |
| Indication of use, N (%) |  |  |  |  |
| Crohn | 7,976 (61.2) | 781 (51.2) | 4,622 (67.9) | 2,573 (54.7) |
| Ulcerative colitis |  |  |  |  |
| Index drug, N (%) |  |  |  |  |
| Adalimumab | 6,398 (49.1) | 383 (25.1) | 4,065 (59.7) | 1,950 (41.4) |
| Golimumab | 567 (4.3) | 84 (5.5) | 295 (4.3) | 188 (4.0) |
| Infliximab | 5,746 (44.1) | 998 (65.4) | 2,280 (33.5) | 2,468 (52.4) |
| Vedolizumab | 330 (2.5) | 60 (3.9) | 170 (2.5) | 100 (2.1) |
| Index route of administration, N (%) |  |  |  |  |
| Intravenous | 6,076 (46.6) | 1,058 (69.4) | 2,450 (36.0) | 2,568 (54.6) |
| Subcutaneous | 6,965 (53.4) | 467 (30.6) | 4,360 (64.0) | 2,138 (45.4) |
| Index class, N (%) |  |  |  |  |
| Selective immunosuppressant | 330 (2.5) | 60 (3.9) | 170 (2.5) | 100 (2.1) |
| Tumor necrosis factor | 12,711 (97.5) | 1,465 (96.1) | 6,640 (97.5) | 4,606 (97.9) |
| Type of index drug, N (%) |  |  |  |  |
| Biosimilar | 1,481 (11.4) | 332 (21.8) | 660 (9.7) | 489 (10.4) |
| Originator | 11,560 (88.6) | 1,193 (78.2) | 6,150 (90.3) | 4,217 (89.6) |
| **Before index date (ever)** | | | | |
| History of comorbidity, N (%) | | | | |
| Hypertension | 1,097 (8.4) | 142 (9.3) | 520 (7.6) | 435 (9.2) |
| Atrial fibrillation | 84 (0.6) | 10 (0.7) | 40 (0.6) | 34 (0.7) |
| Ischemic hearth disease | 188 (1.4) | 33 (2.2) | 82 (1.2) | 73 (1.6) |
| Cerebrovascular disease | 126 (1.0) | 11 (0.7) | 63 (0.9) | 52 (1.1) |
| Diabetes mellitus | 516 (4.0) | 61 (4.0) | 213 (3.1) | 242 (5.1) |
| Chronic renal disease | 110 (0.8) | 9 (0.6) | 55 (0.8) | 46 (1.0) |
| Chronic liver disease | 119 (0.9) | 15 (1.0) | 55 (0.8) | 49 (1.0) |
| Chronic pulmonary disease | 110 (0.8) | 19 (1.2) | 49 (0.7) | 42 (0.9) |
| History of tumors | 504 (3.9) | 54 (3.5) | 261 (3.8) | 189 (4.0) |
| Intestinal infections | 1,488 (11.4) | 146 (9.6) | 833 (12.2) | 509 (10.8) |
| Other IMIDs |  |  |  |  |
| Psoriasis | 583 (4.5) | 66 (4.3) | 335 (4.9) | 182 (3.9) |
| Psoriatic arthritis | 403 (3.1) | 43 (2.8) | 230 (3.4) | 130 (2.8) |
| Rheumatoid arthritis | 348 (2.7) | 37 (2.4) | 186 (2.7) | 125 (2.7) |
| Ankylosing spondylitis | 644 (4.9) | 64 (4.2) | 394 (5.8) | 186 (4.0) |
| **Before index date (one year)** | | | | |
| Previous use of drugs, N (%) | | | | |
| Antiplatelet agents | 44 (0.3) | 4 (0.3) | 20 (0.3) | 20 (0.4) |
| Antiarrhythmics | 8 (0.1) | - | 7 (0.1) | 1 (0.0) |
| Anticoagulants | 446 (3.4) | 64 (4.2) | 251 (3.7) | 131 (2.8) |
| Antidepressants | 131 (1.0) | 6 (0.4) | 83 (1.2) | 42 (0.9) |
| Antipsychotics | 30 (0.2) | 6 (0.4) | 19 (0.3) | 5 (0.1) |
| Antibacterial for systemic use | 67 (0.5) | 12 (0.8) | 38 (0.6) | 17 (0.4) |
| Antivirals for systemic use | 150 (1.2) | 20 (1.3) | 79 (1.2) | 51 (1.1) |
| Drugs for peptic ulcer and gastro-oesophageal reflux disease | 378 (2.9) | 62 (4.1) | 207 (3.0) | 109 (2.3) |
| csDMARDs | 839 (6.4) | 100 (6.6) | 439 (6.4) | 300 (6.4) |
| Methotrexate | 131 (1.0) | 11 (0.7) | 71 (1.0) | 49 (1.0) |
| Sulfasalazine | 177 (1.4) | 19 (1.2) | 91 (1.3) | 67 (1.4) |
| Mesalazine | 545 (4.2) | 75 (4.9) | 281 (4.1) | 189 (4.0) |
| Balsalazide | 14 (0.1) | 3 (0.2) | 6 (0.1) | 5 (0.1) |
| Glucocorticoids | 215 (1.6) | 26 (1.7) | 110 (1.6) | 79 (1.7) |
| NSAIDs | 217 (1.7) | 22 (1.4) | 118 (1.7) | 77 (1.6) |

csDMARDs: conventional synthetic disease-modifying antirheumatic drugs; NSAIDs: nonsteroidal anti-inflammatory drugs

**Table S9**. Distribution of non-biological drugs and biological drugs (switch) users during a 5-year follow-up period, overall and by cluster for a cohort with at least a 5-year follow-up period

|  | **Overall**  **(N=13,041)** | **High adherence**  **(N=1,525)** | **Medium adherence (N=6,810)** | **Low adherence (N=4,706)** |
| --- | --- | --- | --- | --- |
| **After index date (five years)** | | | | |
| Antiplatelet agents, N (%) | 253 (1.9) | 23 (1.5) | 114 (1.7) | 116 (2.5) |
| Antiarrhythmics, N (%) | 62 (0.5) | 5 (0.3) | 18 (0.3) | 39 (0.8) |
| Anticoagulants, N (%) | 1,987 (15.2) | 128 (8.4) | 938 (13.8) | 921 (19.6) |
| Antidepressants, N (%) | 717 (5.5) | 53 (3.5) | 371 (5.4) | 293 (6.2) |
| Antipsychotics, N (%) | 198 (1.5) | 19 (1.2) | 94 (1.4) | 85 (1.8) |
| Antibacterial for systemic use, N (%) | 331 (2.5) | 37 (2.4) | 173 (2.5) | 121 (2.6) |
| Antivirals for systemic use, N (%) | 896 (6.9) | 107 (7.0) | 473 (6.9) | 316 (6.7) |
| Drugs for peptic ulcer and gastro-oesophageal reflux disease, N (%) | 767 (5.9) | 75 (4.9) | 380 (5.6) | 312 (6.6) |
| csDMARDS, N (%) | 2,145 (16.4) | 196 (12.9) | 1,145 (16.8) | 804 (17.1) |
| Balsalazide | 22 (0.2) | 2 (0.1) | 12 (0.2) | 8 (0.2) |
| Mesalazina | 1,076 (8.3) | 105 (6.9) | 568 (8.3) | 403 (8.6) |
| Metotrexato | 714 (5.5) | 58 (3.8) | 422 (6.2) | 234 (5.0) |
| Sulfasalazine | 566 (4.3) | 53 (3.5) | 277 (4.1) | 236 (5.0) |
| Glucocorticoids, N (%) | 704 (5.4) | 74 (4.9) | 393 (5.8) | 237 (5.0) |
| NSAIDs, N (%) | 985 (7.6) | 88 (5.8) | 524 (7.7) | 373 (7.9) |
| Switch, N (%) | 6,222 (47.7) | 624 (40.9) | 3,851 (56.5) | 1,747 (37.1) |
| Time to first switch, median (IQR), days | 838.5  [420.0, 1297.3] | 921.5  [403.0, 1344.0] | 904.0  [519.0, 1303.0] | 511.0  [217.0, 1166.5] |

csDMARDs: conventional synthetic disease-modifying antirheumatic drugs; NSAIDs: nonsteroidal anti-inflammatory drugs; TNF: tumor necrosis factor

**Figure S1**. Study design implementation

**
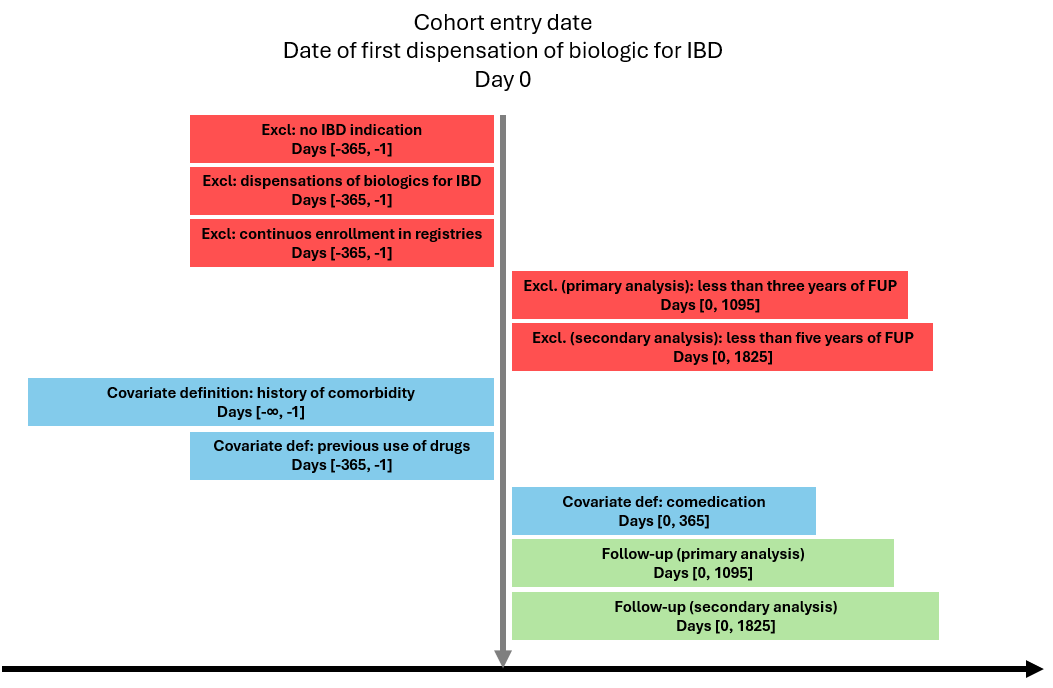
**

**Figure S2**. Trajectories of adherence to biologics for IBDs for a cohort with at least a 5-year follow-up period

**Figure S3**. Determinants of treatment adherence to biologics for a cohort with at least a 5-year follow-up period


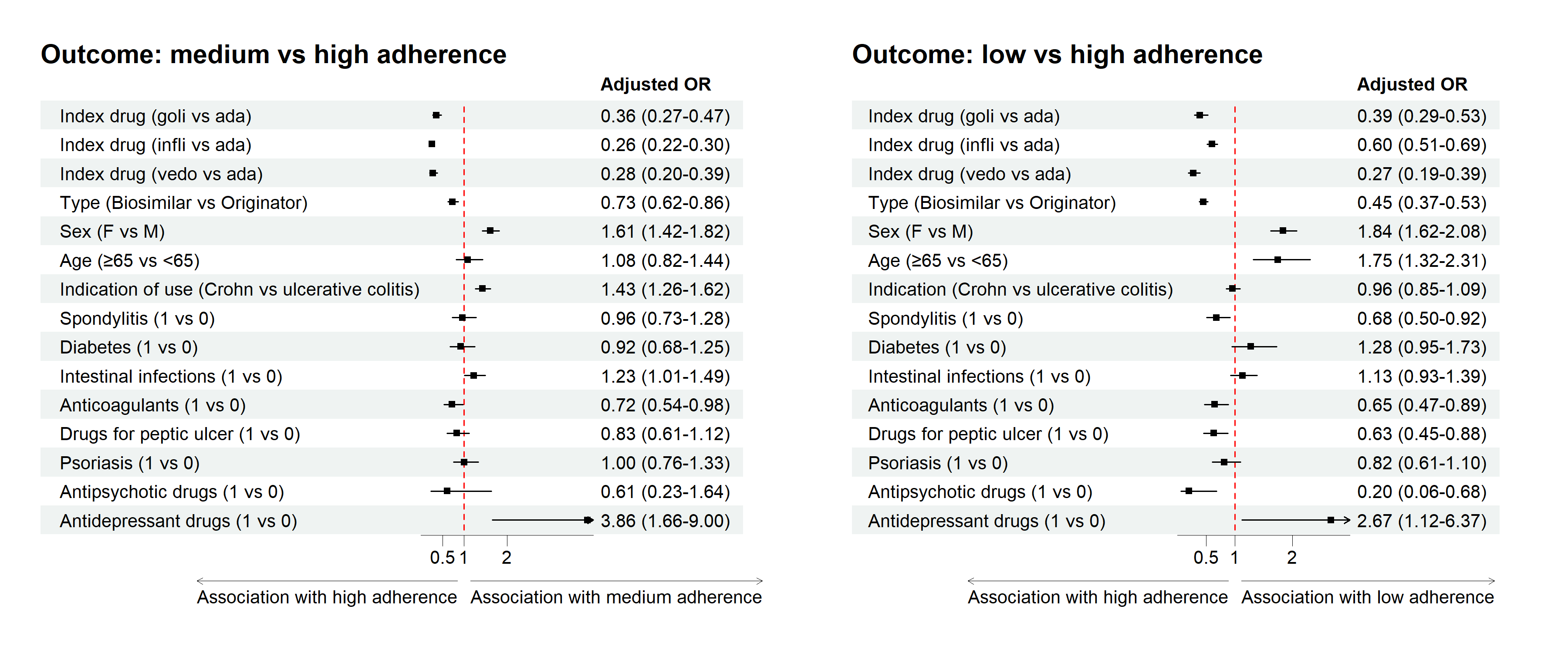

Supplement: Supplementary file 1 — Table S1: Defined daily doses assigned by the WHO. Table S2: Codes used to identify comorbidities. Table S3: Codes used to identify drug therapies. Table S4: Distribution of cohort characteristics at index date and 1 year before, by index drug, for a cohort with at least a 3‐year follow‐up period. Table S5: Distribution of cohort characteristics at index date and 1 year before, by cluster for a cohort with at least a 3‐year follow‐up period. Table S6: Distribution of cohort characteristics at index date and 1 year before for Crohn's disease only, by cluster for a cohort with at least a 3‐year follow‐up period. Table S7: Distribution of cohort characteristics at index date and 1 year before for ulcerative colitis only, by cluster for a cohort with at least a 3‐year follow‐up period. Table S8: Distribution of cohort characteristics at index date and 1 year before, overall and by cluster for a cohort with at least a 5‐year follow‐up period. Table S9: Distribution of non‐biological drugs and biological drugs (switch) users during a 5‐year follow‐up period, overall and by cluster for a cohort with at least a 5‐year follow‐up period. Figure S1: Study design implementation. Figure S2: Trajectories of adherence to biologics for IBDs for a cohort with at least a 5‐year follow‐up period. Figure S3: Determinants of treatment adherence to biologics for a cohort with at least a 5‐year follow‐up period. [file PDS-34-e70230-s001.docx]
